# Relaxin-expressing oncolytic adenovirus induces remodeling of physical and immunological aspects of cold tumor to potentiate PD-1 blockade

**DOI:** 10.1136/jitc-2020-000763

**Published:** 2020-08-04

**Authors:** Bo-Kyeong Jung, Hae Young Ko, Hyunji Kang, JinWoo Hong, Hyo Min Ahn, Youjin Na, Hyeongi Kim, Jin Su Kim, Chae-Ok Yun

**Affiliations:** 1 Department of Bioengineering, College of Engineering, Hanyang University, Seoul, Korea (the Republic of); 2 Division of RI Application, Korea Institute of Radiological and Medical Sciences, Seoul, Korea (the Republic of); 3 Department of Research and Development, GeneMedicine Co., Ltd, Seoul, Korea (the Republic of); 4 Radiological and Medico-Oncological Sciences, University of science and technology (UST), Seoul, Korea (the Republic of); 5 Institute of Nano Science and Technology (INST), Hanyang University, Seoul, Korea (the Republic of)

**Keywords:** immunotherapy, lymphocytes, tumor-infiltrating, oncolytic virotherapy, radioimmunotherapy, tumor microenvironment

## Abstract

**Background:**

Currently, several antibody (Ab)-based therapies have shown excellent therapeutic effects in the clinic. Nonetheless, Ab penetration into tumor tissues is limited due to abnormal vasculature, tumor interstitial pressure, and excessive extracellular matrix (ECM) accumulation, thus demanding novel strategies to overcome these barriers.

**Methods:**

The intratumoral distribution of therapeutic Abs were detected by fluorescence microscopy or positron emission tomography in both human gastric xenograft and syngeneic pancreatic hamster tumor models. The antitumor efficacy by combination of oncolytic adenovirus (Ad), which coexpresses relaxin (RLX), interleukin (IL)-12, and granulocyte macrophage colony-stimulating factor (GM-CSF) (oAd/IL12/GM-RLX) and antibody against the programmed cell death protein 1 (αPD-1) was examined in hamster subcutaneous and orthotopic pancreatic tumor models. The immunological aspects of these combination therapy regimen were assessed by flow cytometry or immunohistochemistry in subcutaneous hamster tumor models.

**Results:**

Relaxin-expressing oncolytic Ad effectively degraded tumor ECM and enhanced the tumor penetration of trastuzumab in comparison with trastuzumab monotherapy. Based on these results, an oAd/IL12/GM-RLX was used to enhance the potency of immune checkpoint blockade. The combination of the oAd/IL12/GM-RLX and αPD-1 promoted a concomitant degradation of the tumor ECM and amelioration of the immunosuppressive tumor niches, ultimately enhanced intratumoral infiltration of both αPD-1 and activated T cells. Of note, the combination therapy was able to elicit a potent and durable antitumor immune response against cold tumors that were refractory to immune checkpoint inhibitor monotherapy.

**Conclusions:**

Our findings are the first to demonstrate that expression of four genes (IL-12p35, IL-12p40, GM-CSF, and RLX) mediated by a single oncolytic Ad vector can promote remodeling of both physical and immunological aspects of the tumor niches to overcome the major limitations of Ab-based therapies that have emerged in recent clinical trials.

## Background

Antibody (Ab)-based cancer therapy has been one of the most effective strategies for treating patients with tumors. More than 20 products have been approved by the US Food and Drug Administration for cancer, and other products are investigated in clinical trials.^1^ The mechanisms of tumor cell killing by therapeutic Abs include a direct action of the Ab and immune response-mediated cell killing mechanisms.^2^

Several tumor microenvironmental factors, which include an abnormal vasculature, elevated interstitial pressure, high cell density, and dense extracellular matrix (ECM), function as the primary barriers against drug penetration.^3^ Tumor-derived ECM, which consists of a dense network of various collagens, fibronectins, and proteoglycans, inhibits intratumoral penetration and dispersion of therapeutics.^4^ Thus, determining strategies to breach the ECM is an important goal.

Preferential transfer of a gene encoding an ECM-degrading protein to tumor by viral vectors could induce durable ECM degradation. Among several vectors in development for cancer therapy, oncolytic adenovirus (Ad) is particularly promising due to proven safety record of Ad in numerous clinical trials, preferential cytolytic activity against tumor, no risk of insertional mutagenesis, high gene transfer efficacy, and cancer-specific and transient therapeutic gene expression that is amplified with each replication cycle and secondary infection of adjacent cancer cells.^5^ oAd without ECM-degrading transgene cannot be efficiently dispersed away from the injection site in tumor tissues due to dense tumor ECM; thus, we have employed relaxin (RLX) as one of the therapeutic transgene in present report due to its ability to inhibit the de novo ECM synthesis and to decompose the overexpressed ECM components in diseases with aberrant fibrosis (eg, keloid and desmoplasia in tumor), as well as improving viral dispersion in tumor tissues.^6^ Further, we hypothesized that ECM degradation by RLX-expressing oAd will also aid the intratumoral penetration and dispersion of therapeutic Abs.

Among therapeutic Abs, immune checkpoint inhibitors (ICIs), such as antibody against the programmed cell death protein 1 (αPD-1) or anti-cytotoxic T-lymphocyte antigen 4 Abs, have been extensively evaluated in various phases of clinical development for their ability to elicit potent antitumor immune response.^7^ Although ICI treatments can lead to durable responses against multiple types of cancers in a subset of patients, they are ineffective in patients harboring ‘cold’ tumors in which the level of immune cell infiltration is low.^8 9^ In this regard, the development of rationally designed combination therapy strategies is necessary to improve the effect of ICIs by facilitating CD4^+^ and CD8^+^ T-cell recruitment and activation in the tumor microenvironment.^10^ Oncolytic viruses encoding tumor antigens, cytokines, and/or costimulatory molecules could be a promising means of enhancing ICI activity in ‘cold’ tumors, with numerous reports demonstrating that oncolytic viruses boost antitumor immune responses.^11^

In this study, we used RLX-expressing and immunostimulatory cytokine-expressing oncolytic Ads to enhance the penetration and immune response mediated by an ICI against immunosuppressive pancreatic tumors, which are extremely resistant toward ICI due to desmoplasia, restricting intratumoral infiltration of T cells.^12^ First, we demonstrate that a relaxin-expressing oncolytic adenovirus (oAd/RLX) increases the intratumoral distribution and penetration of trastuzumab by promoting the degradation of ECM in a gastric cancer xenograft model. Building on these initial results, we combined αPD-1 with an immune stimulatory and ECM degrading oncolytic Ad, which coexpresses interleukin (IL)-12, granulocyte macrophage colony-stimulating factor (GM-CSF), and RLX, to elicit potent and durable antitumor immune responses against pancreatic cancer. Importantly, triple therapeutic gene expression mediated by oncolytic Ad enhances the intratumoral distribution of αPD-1 Ab and concomitantly boosts the antitumoral immune response through improved intratumoral infiltration of activated T cells, demonstrating that oncolytic Ad-mediated remodeling of both physical and immunological aspects of the tumor microenvironment is a promising strategy for enhancing the potency of ICI against poorly immunological solid tumors.

## Materials and methods

### Cell lines and cell culture

The human gastric cancer cell line NCI-N87 was cultured in RPMI-1640 medium (GIBCO-BRL, Grand Island, New York, USA). The hamster pancreatic carcinoma cell line HaP-T1 and luciferase-expressing HP-1 (HP-1/Fluc) were cultured in Dulbecco’s modified Eagle’s medium (GIBCO-BRL). All growth medium was supplemented with 10% fetal bovine serum (FBS; GIBCO-BRL), 1% penicillin–streptomycin (100 U/mL). All cells were maintained at 37°C in a humidified atmosphere with 5% CO_2_. The NCI-N87 cell line was purchased from American Type Culture Collection (Manassas, Virginia, USA); HaP-T1 cell line was kindly provided by Dr Masato Abei (University of Tsukuba, Ibaraki, Japan); and HP-1/Fluc, which stably express firefly luciferase, was generated using a lentiviral vector, using the HP-1 cell line provided by Dr Masato Yamamoto (University of Minnesota, Minneapolis, Minnesota).

### Virus preparation

The construction, generation, and characterization of oAd/RLX have been reported previously.^13^ To generate oAd/IL12/GM-RLX, we first constructed a pSP72-E3 Ad E3 shuttle vector expressing GM-CSF and RLX (pSP72-E3/GM-RLX). The pSP72-E3/GM-RLX vector was constructed by subcloning the GM-CSF–Internal ribosome entry site (IRES) PCR product into the previously generated pSP72-E3/RLX vector.^13^ The newly constructed pSP72-E3/GM-RLX shuttle vector was linearized with *Xmn*I digestion, and pAd-ΔB7/IL-12, an Ad-based E1B/E3-deleted total vector with a substitution in the retinoblastoma binding sites of E1A, which expresses IL-12 in the E1 region of Ad-ΔB7,^14^ was linearized with *Spe*I digestion. The linearized pSP72-E3/GM-RLX E3 shuttle vector was then cotransformed into *Escherichia coli* BJ5183, along with the *Spe*I-digested pAd-ΔB7/IL-12 for homologous recombination, resulting in the pAd-ΔB7/IL-12/GM-CSF–IRES-RLX Ad vector. To produce the corresponding Ad, purified plasmids were digested with *Pac*I and transfected into 293A cells, a human embryonic kidney cell line expressing the Ad E1 region, to generate oAd-ΔB7/IL-12/GM-CSF–IRES-RLX (oAd/IL12/GM-RLX). All Ads were propagated in 293A cells and purified by CsCl gradient centrifugation. The number of viral particles (VPs) was determined by measuring the optical density at 260 nm, for which an absorbance value of 1 is equivalent to 1.1×10^12^ VP/mL.

### Preparation of Alexa Fluor 488-conjugated Ab

A solution (10 mM) of Alexa Fluor 488 (Invitrogen, Grand Island, New York, USA) was dissolved in dimethyl sulfoxide with 1% acetic acid. The solution was mixed with 5 mg of Trastuzumab (TZB; Roche, Basel, Switzerland) or αPD-1 (clone RMP1-14; Bio X Cell, West Lebanon, New Hampshire, USA) in 250 µL of 1 M sodium bicarbonate solution, pH 8.5 and allowed to stand for 1 hour at room temperature. The Alexa Fluor 488-conjugated Ab was purified with a size exclusion PD-10 column (GE Healthcare Bio-Sciences AB, Uppsala, Sweden). The number of Alexa Fluor 488 molecules conjugated per Ab was estimated by determining the Alexa Fluor 488 peak intensity distribution between the Ab-Alexa Fluor 488 conjugate and the free Alexa Fluor 488 eluted from the size-exclusion HPLC column (Waters Corporation, Milford, Massachusetts, USA).

### Assessment of trastuzumab distribution in tumor tissue

Nude mice were subcutaneously inoculated with 5×10^6^ NCI-N87 cells. When the average tumor volume reached 200 mm^3^, tumor-bearing mice were intravenously administered with phosphate-buffered saline (PBS), Alexa 488-conjugated TZB (488-TZB; 150 µg), or oAd/RLX (2.5×10^10^ VP) plus 488-TZB (150 µg). The first day of treatment was designated as day 0. oAd/RLX was administered three times in total, whereas a single dose of 488-TZB was administered. On the fifth day after the last administration, 1 mg of rhodamine–lectin (rhodamine ricinus communis agglutinin I) was intravenously injected for visualization of blood vessels. Tumors were harvested with intact skin and flash-frozen using liquid nitrogen for subsequent sectioning and staining. Tumor sections were fixed with 4% paraformaldehyde for 10 min and mounted with Prolong Gold antifade reagent with 4,6-diamidino-2-phenyindole (DAPI) (Invitrogen, Carlsbad, California, USA).

### Acquisition and analysis of fluorescent images

Imaging was performed with a ×10 objective lens using a fluorescent microscope (IN Cell Analyzer, GE Healthcare, Waukesha, Wisconsin, USA) and equipped with mosaic stitching software (IN Cell developer toolbox, GE Healthcare). Three independent channels were obtained: DAPI for nuclei (blue), rhodamine for blood vessels (red), and Fluorescein isothiocyanate (FITC) for 488-TZB (green). The pixel size was 0.65 µm/pixel.

Values were pooled together from 40 regions of each sample to represent the tumor. Each tumor was treated as an independent sample (n=10). To measure the total uptake of TZB, the total area of green intensity in the tumor was divided by the total tumor area. To calculate the vascular density, an individual blood vessel image was segmented using the fuzzy c-means clustering method and converted to a binary image. The fraction of vessel area over the entire tumor area was determined to obtain the vascular density (%).

Because Ab penetrated into the tissues from both tumor surface and blood vessels,^15^ 20-line profiles were plotted from tumor surface and blood vessel (0–150 µm) on 10 tumor samples. Each tumor was treated as an independent sample (n=10). All line profiles from each group were analyzed using an in-house program written in MATLAB (MathWorks, Natick, Massachusetts, USA). The line profile intensity was averaged using the following equation:

$$I\left(x_{i}\right)=\frac{1}{N_{ij}}\sum_{j=1}^{N_{j}}\sum_{n}\phi_{ni}\left(a_{jn}\right)\times f_{j}\left(a_{jn}\right)$$

where $N_{j}$=the number of samples (the number of line profiling),

$$a_{jn}\in lineprofilingdata\left(j^{\prime }ssample\right)$$

$$a_{jn}\in x_{j}=lineprofilingdata\left(j^{\prime }ssample\right)$$

$$f_{j}\left(x\right)=lineprofilingintensitydata\left(j\prime ssample\right)$$

$$\Delta x_{i}=\left[x_{i}-\frac{h}{2}+h\times \left(i-1\right),x_{i}+\frac{h}{2}+h\times \left(i-1\right)\right]$$

$$for\forall_{j}N_{ij}=n(\sum_{j}(x_{j}\cap \Delta_{xi}))$$

To identify the difference in Ab uptake, the area under the curve (AUC) was quantified. AUC was represented as mean±SD.

### Assessment of antitumor efficacy by combination therapy of RLX-expressing oncolytic AD and αPD-1

Syrian golden hamsters (Japan SLC, Tokyo, Japan) were maintained in a laminar airflow cabinet under specific pathogen-free conditions. All facilities were approved by the Association and Accreditation of Laboratory Animal Care.

For the subcutaneous tumor model, tumors were implanted subcutaneously on the right flank of Syrian golden hamsters by inoculating 3×10^6^ HaP-T1 cells suspended in 50 µL of Hank’s balanced salt solution (GIBCO-BRL). To assess the antitumor effect of various treatments, oAd/IL12/GM-RLX and αPD-1, PBS, αPD-1 (10 mg/kg), oAd/IL12/GM-RLX (7×10^7^ or 1×10^9^ VP), or oAd/IL12/GM-RLX (7×10^7^ or 1×10^9^ VP) plus αPD-1 (10 mg/kg) were administered to the tumor-bearing hamsters. The first day of treatment was designated as day 0. To assess the antitumor efficacy of each therapeutic in ICI refractory tumors, αPD-1 (10 mg/kg) was administered at days 2, 5, and 8 to generate ICI refractory tumors, which were subsequently administered four times with oAd/IL12/GM-RLX (1×10^9^ VP) at intervals of 2 days from day 9. All treatments began when the average tumor volume was approximately 100 mm^3^. All treatments except αPD-1, which was administered via intraperitoneal injection, were administered intratumorally. Tumor growth was evaluated every day by taking measurements of the length (L) and width (W) of the tumor. Tumor volume was calculated using the following formula: volume=0.523 L(W)^2^.

For the orthotopic tumor model, HP-1 cancer cells (4×10^5^) were injected directly into the tail of pancreas, and the establishment of the orthotopic pancreatic tumor model was confirmed by bioluminescence imaging as previously reported.^16^ HP-1 orthotopic pancreatic tumor-bearing hamsters were administered 1×10^9^ VP of oAd/IL12/GM-RLX into the abdomen twice (days 4 and 6), followed by a single injection into the tumor (day 8). αPD-1 was injected intraperitoneally at 3 day intervals three times (days 6, 9, and 12). The tumor volume was measured by luciferase signal at days 4, 9, and 13 after cancer cell injection.

### Histological and immunohistochemical analysis

For histological analysis, tumor tissues were collected from hamsters at 10 days postinitial Ad injection, embedded in paraffin, and sectioned at 4 µm thickness for H&E staining. For immunohistochemical staining, the 4 µm-thick tumor sections were blocked with 3% bovine serum albumin in Tris-buffered saline (Sigma, St. Louis, Missouri, USA) for 2 hours. The tumor sections were then incubated with a mouse anticollagen type I Ab (Cell Signaling Technology, USA) or a mouse antiproliferating cell nuclear antigen (PCNA) Ab (M0879; DAKO, Glostrup, Denmark) as primary Ab. After washing, the sections were incubated with goat anti-mouse IgG Ab (H+L)-HRP (1031–05; Southern Biotech) as a secondary Ab, and then counterstained with Meyer’s hematoxylin (Sigma). A terminal deoxynucleotidyl transferase dUTP nick end labeling assay was performed as previously described.^16^ To identify lymphocyte infiltration into tumor tissues, tumor tissues were frozen in OCT compound (Sakura Finetec, Torrance, California, USA) and cut into 8 µm sections. Tumor sections were fixed with chilled acetone for 10 min and blocked with Blocking Solution (DAKO). Sections were then incubated with primary Abs, mouse anti-rat CD4 monoclonal Ab (ebioscience, San Diego, California, USA) and mouse anti-rat CD8 monoclonal Ab (ebioscience) at 4°C overnight. After washing three times with PBS, samples were incubated with the secondary Ab, Alexa 647 goat anti-mouse Ab (Abcam, Cambridge, Massachusetts, USA) at room temperature for 2 hours. In the final step, the slides were washed with PBS, mounted with Prolong Gold antifade reagent with DAPI (Invitrogen), and then examined under a fluorescence microscope (IN Cell analyzer 2200).

### Preparation of radiolabeled Ab

^64^Cu is a radionuclide produced by a cyclotron with an intermediate half-life (t_1/2_, 12.7 h) that decays by both β+ (655 keV, 17.4%) and β– (573 keV, 39.0%) emission, making it suitable for both labeling Abs for positron emission tomography (PET) imaging of cancer.^17^ 1,4,7,10-Tetraazacyclododecane-1,4,7,10-tetraacetic acid (DOTA)-conjugated Ab (DOTA-Ab) was prepared by conjugating DOTA-NHS-ester (Macrocyclics, Dallas, Texas, USA) to 10 mg of Ab with a 20-fold molar ratio in 0.1 M sodium bicarbonate buffer, pH 8.5. The mixtures were incubated overnight with mild shaking at 4°C, after which the conjugated Abs were purified from excess DOTA-NHS-ester with a PD-10 size exclusion column (GE Healthcare) using 1 mM sodium acetate buffer, pH 6.5. ^64^CuCl_2_ was produced by 50 MeV cyclotron irradiation at the Korea Institute of Radiologic and Medical Sciences.^18^ DOTA-Ab (3 mg) was incubated with ^64^Cu (370 MBq) in 1 mM sodium acetate buffer, pH 6.5, for 1 hour at 37°C. After incubation, ^64^Cu-conjugated DOTA-Ab was purified by use of a PD-10 size exclusion column that was eluted with 1 mM sodium acetate buffer. The radiochemical purity of ^64^Cu-Ab was ≥98% by analysis with thin layer chromatography. The immunoreactivity of ^64^Cu-TZB or ^64^Cu-αPD-1 was determined to be ≥93% using a cell-binding assay.

### ImmunoPET imaging

When the average tumor size in NCI-N87 tumor-bearing mice reached 200 mm^3^, ^64^Cu-TZB (150 µg, 14.8–18.5 MBq/200 µL) was administered on day 0, whereas oAd/RLX (2.5×10^7^ VP) was administered on days 0, 2, and 4 via intravenous injection. For imaging of ^64^Cu-αPD-1, HaP-T1 tumor-bearing hamsters were intratumorally treated with oAd/IL12/GM-RLX (7×10^7^ VP) on days 0 and 2, whereas ^64^Cu-αPD-1 (400 µg, 17.39–20.35 MBq/200 µL) was intraperitoneally injected on day 2. Before PET imaging with a Siemens Inveon PET scanner (Siemens Healthcare, Erlangen, Germany), mice or hamsters were anesthetized with 2.5% isoflurane. For the acquisition of anatomical images, CT imaging was acquired with the second bed position, full rotation, and 180 projections per bed position. The exposure time was 200 ms and the estimated scan time was 504 s for CT imaging. CT data were reconstructed using Feldkamp reconstruction with a Shepp-Logan filter. The effective pixel size of the reconstructed CT image was 109.69 μm×109.69 μm. PET data were acquired for 15 min within an energy window of 350–650 keV at each time point after injection of ^64^Cu-TZB and ^64^Cu-αPD-1. The PET data were reconstructed with a 3D reprojection algorithm for regions of interest (ROI) analysis. The matrix size was 128×128×159, and the voxel size was 0.776×0.776×0.796 mm^3^. PET and CT images were coregistered using Inveon Research Workplace V.2.0 (Siemens Healthcare). After coregistration of CT and PET data, the ROI was drawn on the CT image and copied to the PET data. %ID/g was calculated by C_T_⋅(V_T_/W_T_)⋅(1/D_inj_)⋅100%, where D_inj_ is the injected dose (mCi); C_T_ is the radioactivity in the tissue region (mCi/cc); W_T_ is weight; and V_T_ represents volume. Counts/second/voxel values were obtained from a PET ROI, and mCi/cc tissue was calculated using a cylinder calibration factor. The density of tissue was assumed to be ~1 cc tissue/g of tissue.

### Autoradiography

Immediately after PET/CT scanning, tumor tissues were isolated and frozen in OCT compound. After decaying for 48 hours, frozen tumors were sectioned at 20 µm thickness using a cryostat microtome and exposed on an imaging plate for 24 hours. The plates were scanned with a BAS-5000 (Fujifilm, Tokyo, Japan). The signal intensities after tumor uptake of ^64^Cu-TZB or ^64^Cu-TZB plus oAd/RLX were quantified as units of photostimulated luminescence per square millimetre (PSL/mm^2^) using Multi Gage software V.3.0 (Fujifilm). For histological analysis, tumor tissues were stained by H&E and viewed under a light microscope (Carl Zeiss, Oberkochem, Germany).

### Biodistribution study

After PET scanning at 60 hours after administration of ^64^Cu-αPD-1, hamsters (n=3, each group) were euthanized by CO_2_ gas asphyxiation and exsanguinated by cardiac pucture before dissection. The organs, tumor, and blood were collected and weighed. The decay-corrected radioactivity of the organs, tumor, and blood was measured as the total number of counts using a Wizard2 gamma-counter (Perkin Elmer, Waltham, Massachusetts, USA). The activity data were represented as the percentage injected radioactivity dose per gram of tissue (%ID/g).

### Fluorescence-activated cell sorting (FACS) analysis

HaP-T1 tumor-bearing hamsters were intratumorally injected with PBS, αPD-1 (10 mg/kg), oAd/IL12/GM-RLX (7×10^7^ VP), or oAd/IL12/GM-RLX (7×10^7^ VP) plus αPD-1 (10 mg/kg). At 12 days after the initial treatment, lymphocytes were isolated from draining lymph node (DLN) or tumor tissue as previously reported.^19^ Before staining, cells were treated with saturating anti-CD16/CD32 (Biolegend) in staining buffer (2% FBS and 0.02% sodium azide in PBS). Then, cells were stained with hamster anti-mouse CD3e monoclonal Ab (BD Bioscience, San Jose, California, USA), mouse anti-rat CD4 monoclonal Ab (ebioscience), or mouse anti-rat CD8 monoclonal Ab (ebioscience) for the assessment of the CD4 or CD8 and interferon (IFN)-γ coexpressing T-cell population. After staining of surface markers (CD3, CD4, and CD8), cells were fixed and permeabilized with Cytofix/Cytoperm solution (BD pharMingen, San Jose, California, USA) and then stained with a rabbit anti-hamster IFN-γ Ab (Abclon, Seoul, Korea). Samples were analyzed using a FACScan flow cytometer with CellQuest software (Beckton-Dickinson) as previously reported.^19^

### IFN-γ ELISA

Lymphocytes were harvested from PBS-treated, αPD-1-treated, oAd/IL12/GM-RLX-treated or oAd/IL12/GM-RLX plus αPD-1-treated hamsters and minced into single-cell suspensions in PBS containing 2% FBS. The lymphocytes were then cocultured with irradiated HaP-T1 cells for 3 days in the presence of recombinant human IL-2 (Peprotech, Rocky Hill, New Jersey, USA). The supernatants were collected, and an IFN-γ ELISA was carried out according to the manufacturer’s protocol (Cusabio Biotech, Wuhan, China).

### Statistical analysis

All statistical analyses were performed by two-tailed Student t-test or one-way analysis of variance (SPSS 13.0 software; SPSS, Chicago, Illinois, USA). Data are expressed as mean±SD. P values less than 0.05 were assumed to denote statistical significance.

## Results

### Characterization of oAd/RLX

Because dense and abnormal ECM functions as a physical barrier against drug dispersion, an oncolytic Ad expressing RLX (oAd/RLX, online supplementary figure S1A) was constructed to degrade tumor ECM. oAd/RLX expresses RLX in a dose-dependent manner (online supplementary figure S1B), decreases the accumulation of ECM in tumor tissues, and lowers the expression level of collagen by 80% compared with PBS-treated group (online supplementary figure S1C–E, ***p<0.001).

10.1136/jitc-2020-000763.supp1 Supplementary data

To assess whether systemically administered oAd/RLX causes degradation of ECM in other organs, articular cartilage was isolated from oAd/RLX-treated and PBS-treated mice, and the proteoglycan matrix was stained with Safranin-O (online supplementary figure S2). oAd/RLX (2.5×10^7^ VP, three times) had no adverse side effect on the proteoglycan matrix of fibrocartilage in the knee joint.

### Enhanced penetration of trastuzumab following oAd/RLX treatment

**Figure 1:**
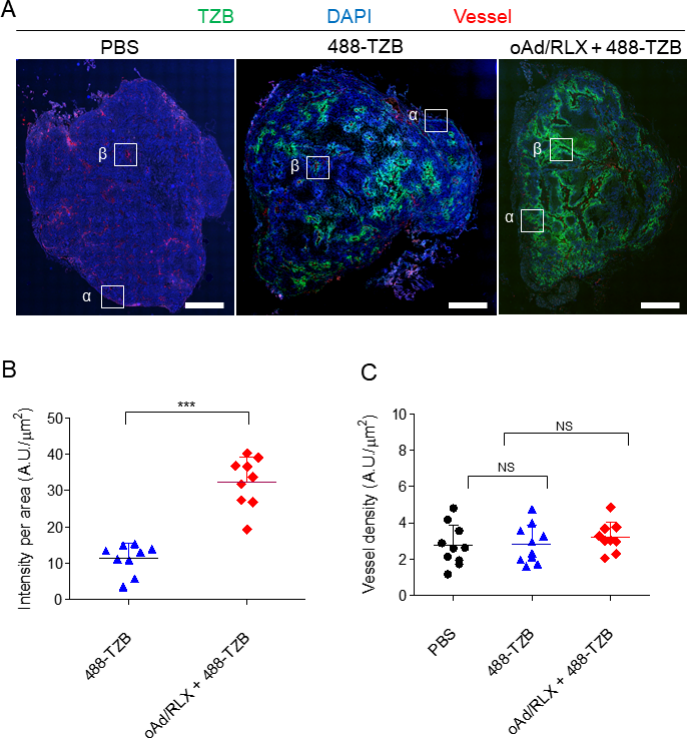
Distribution of TZB in whole tumor tissue. (A) Fluorescence image of 488-TZB (green) in NCI-N87 xenografted tumors. Tumors were isolated at 5 days after injection of 488-TZB or oAd/RLX plus 488-TZB into NCI-N87-xenograft mice. Blood vessels (red) and nuclei (blue) are shown for each group. The white boxes correspond to the regions displayed in figure 2A and online supplementary figure S3A. The scale bar represents 1 mm. (B) The total accumulation of 488-TZB per tumor tissue area. The total accumulation of 488-TZB in the whole tumor region was measured from whole tissue fluorescence images (four images per mouse, mouse n=10). 488-TZB versus oAd/RLX+488 TZB, ***p<0.001, statistical analyses were performed by two-tailed Student t-test. (C) The density of rhodamine–lectin-positive functional blood vessels. The vascular density was measured using the red signals from rhodamine–lectin-stained functional blood vessel images (four images per mouse, mouse n=10). To calculate the vascular density, an individual blood vessel image was segmented using the fuzzy C-means clustering method and converted to a binary image. The fraction of vessel area over the entire tumor area was determined to obtain the vascular density (%). Quantitative data are presented as mean optical density±SD (n=10). TZB, trastuzumab; DAPI, 4,6-diamidino-2-phenyindole; PBS, phosphate-buffered saline; 488-TZB, Alexa 488-conjugated trastuzumab; NS, not significant; oAd/RLX, relaxin-expressing oncolytic adenovirus.

determine whether oAd/RLX enhances the intratumoral penetration and accumulation of Ab, tumor tissues from mice treated with Alexa 488-conjugated TZB (488-TZB) and/or oAd/RLX were isolated at 5 days after injection, and the localization of 488-TZB was observed. As shown in figure 1A, the quantity of 488-TZB was significantly increased in the oAd/RLX plus 488-TZB-treated tumors as compared with the 488-TZB-treated group. We further investigated 488-TZB accumulation in whole tumor tissues by analyzing fluorescence images. The accumulation of 488-TZB per tissue area was increased 2.9-fold in the 488-TZB-treated group compared with the oAd/RLX plus 488-TZB-treated group (figure 1B, ***p<0.001). We next determined if the increased Ab accumulation was associated with a change in blood vessel density in tumor tissues. As shown in figure 1C, the functional blood vessel density was not significantly changed after oAd/RLX treatment. These results suggest that oAd/RLX enhances the accumulation and penetration of therapeutic Abs in tumor tissue by inducing ECM remodeling in the tumor microenvironment, without changing the blood vessel density.

### Enhanced distribution of trastuzumab from the tumor edge and blood vessels induced by oAd/RLX

**Figure 2:**
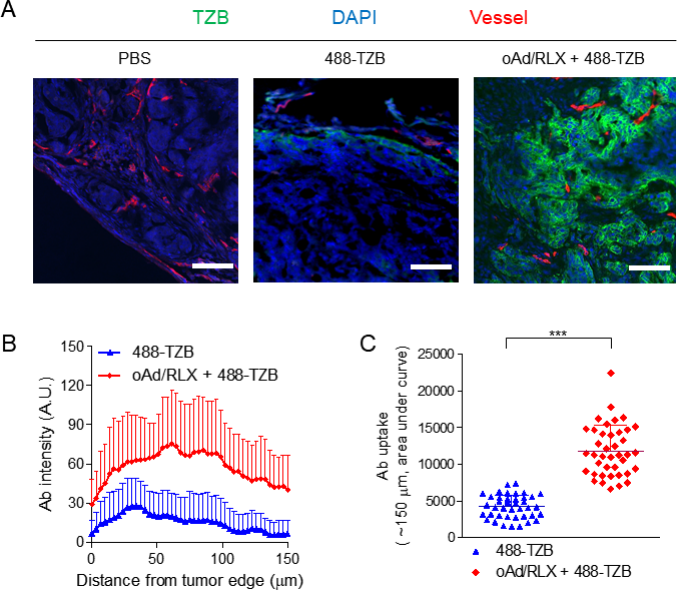
In vivo accumulation and penetration of 488-TZB relative to the tumor edge and functional blood vessels. (A) Fuorescence images magnified from the white boxes labeled α in figure 1A. 488-TZB (green), rhodamine-lectin-positive functional blood vessels (red), and DAPI-stained nuclei (blue) are shown for each group. The scale bar represents 100 µm. (B) Quantitative analysis of 488-TZB intensity in tumor tissue (0–150 µm from the tumor edge). (C) Uptake of 488-TZB quantified by area under curve analysis (0–150 µm from the tumor edge). Quantitative data are presented as mean optical density±SD (n=10); 488-TZB versus oAd/RLX+488 TZB, ***p<0.001. Statistical analyses were performed by two-tailed Student t-test. Ab, antibody; TZB, trastuzumab; DAPI, 4,6-diamidino-2-phenyindole; PBS, phosphate-buffered saline; 488-TZB, Alexa 488-conjugated trastuzumab; oAd/RLX, relaxin-expressing oncolytic adenovirus.

To determine how oAd/RLX affects the tumor infiltration of 488-TZB, we plotted line profiles indicating the distribution of 488-TZB relative to the tumor edge on various layers of the fluorescence images (figure 2A). The infiltration depth of 488-TZB was greatest in the oAd/RLX plus 488-TZB group; the largest fraction of 488-TZB accumulation occurred at 61.2 µm from the tumor surface and the accumulation decreased in a distance-dependent manner (figure 2B). Importantly, improved penetration of therapeutic Abs led to enhanced Ab accumulation in tumor tissues in the oAd/RLX plus 488-TZB-treated group, resulting in 2.8-fold greater accumulation than in tumor tissues treated with 488-TZB alone (figure 2C, ***p<0.001).

The intensity profile of 488-TZB was analyzed relative to blood vessels within tumor tissue (online supplementary figure S3A). 488-TZB penetrated deeper into blood vessels when used in conjunction with oAd/RLX than did 488-TZB alone, resulting in 3.2-fold greater 488-TZB accumulation in the blood vessel (online supplementary figure S3B, C, ***p<0.001). These findings suggest that the degradation of tumor stromal components by oAd/RLX could improve tissue penetration of therapeutic Abs, resulting in enhanced drug distribution further away from the tumor edge and blood vessels.

### Increased accumulation of trastuzumab in tumor tissues induced by oAd/RLX

**Figure 3:**
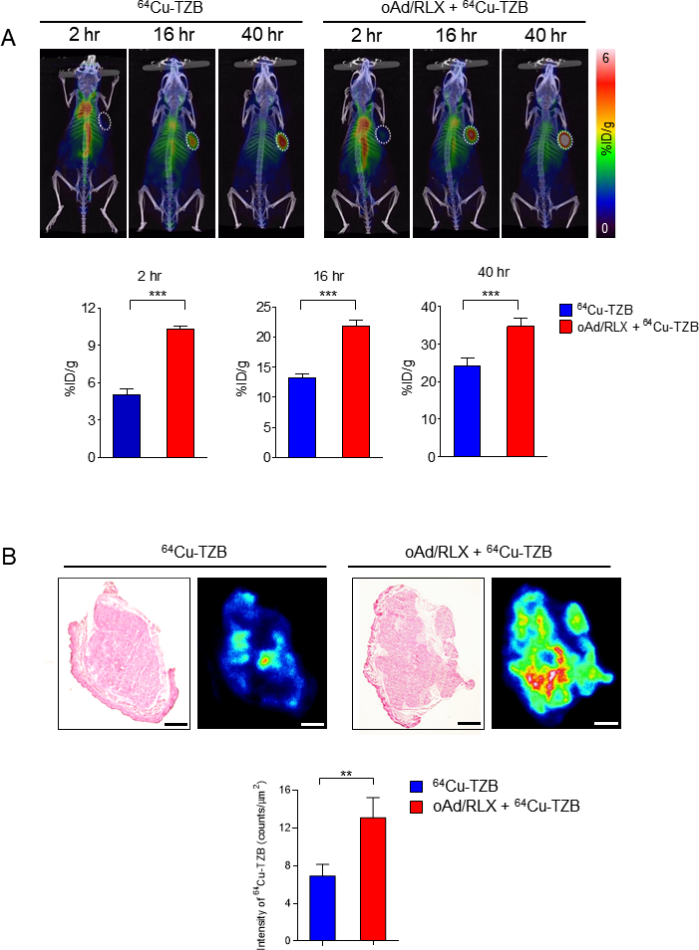
ImmunoPET images. (A) Representative PET/CT images of NCI-N87 tumor-bearing mice at 2, 16, and 40 hours after injection of ^64^Cu-TZB alone (left) or oAd/RLX plus ^64^Cu-TZB (right). White dotted circles indicate the tumor regions. Quantitative data are presented as mean±SD (n=5); ^64^Cu-TZB versus oAd/RLX plus ^64^Cu-TZB, ***p<0.001. Statistical analyses were performed by by two-tailed Student t-test. (B) Quantification of the intratumoral distribution of ^64^Cu-TZB in NCI-N87 tumors by H&E staining and autoradiography. After acquisition of PET images, tumors were isolated from mice. Tumor sections were exposed to an image plate for scanning. The scale bar represents 1 mm. Quantitative data are presented as mean optical density±SD (n=3); ^64^Cu-TZB versus oAd/RLX plus ^64^Cu-TZB, **p<0.01. Statistical analyses were performed by two-tailed Student t-test. TZB, trastuzumab; oAd/RLX, relaxin-expressing oncolytic adenovirus; PET, positron emission tomography.

To evaluate the penetration of Ab into tumors, PET imaging of ^64^Cu-DOTA-TZB (^64^Cu-TZB) and/or oAd RLX injected mice was conducted. As shown in figure 3A, higher uptake of ^64^Cu-TZB was observed in tumors treated with a combination of oAd/RLX plus ^64^Cu-TZB than in those treated with ^64^Cu-TZB at all time (***p<0.001). Autoradiography showed a greater tumor uptake of ^64^Cu-TZB in the oAd/RLX plus ^64^Cu-TZB-treated group than in the ^64^Cu-TZB-treated group (figure 3B, **p<0.01). The enhancement in ^64^Cu-TZB accumulation was tumor-specific because radioisotope accumulation was similar in normal tissues in the ^64^Cu-TZB-treated group and oAd/RLX plus ^64^Cu-TZB-treated groups (online supplementary table S1). Together, these results suggest that ^64^Cu-TZB can be used safely in combination with oAd/RLX with minimal off-target toxicity.

### Potent therapeutic efficacy of the IL-12, GM-CSF, and RLX-coexpressing oncolytic Ad in combination with αPD-1 in a Syrian hamster tumor model

We have designed and constructed an oncolytic Ad coexpressing antitumor cytokines (IL-12 and GM-CSF) and RLX (oAd/IL12/GM-RLX; online supplementary figure S4A), ultimately aiming to restore antitumor immune functions in the tumor milieu and enhance the intratumoral penetration of the oncolytic Ad, ICIs, and immune cells through ECM degradation. The expression of three therapeutic genes by oAd/IL12/GM-RLX increased in a dose-dependent manner (online supplementary figure S4B). These results demonstrate that single vector arming three functional therapeutic genes can express all therapeutic genes effectively in cancer cells. Next, we analyzed whether the combined expression of three therapeutic genes translate to more potent antitumor effect than oncolytic Ad expressing either RLX alone (oAd/RLX)^13 20^ or coexpressing IL-12 and GM-CSF (oAd/IL12/GM)^21^ in a Syrian hamster syngeneic pancreatic tumor model. As shown in online supplementary figure S9, all therapeutic gene expressing oAds (oAd/RLX, oAd/IL12/GM, and oAd/IL12/GM-RLX) elicited more robust tumor growth inhibition than control oAd. Antitumor cytokine expressing oAds (oAd/IL12/GM and oAd/IL12/GM-RLX) elicited more potent antitumor effect than oAd or oAd/RLX, in line with reports showing that immune boosting genes are integral to maximizing antitumor effect of oncolytic viruses in field of immuno-oncology. Importantly, oAd/IL12/GM-RLX had a more potent antitumor effect than oAd/IL12/GM (***p<0.001), showing that addition of RLX improved overall potency of oAd (online supplementary figure S9A). In detail, oAd/IL12/GM-RLX induced higher intratumoral expression of E1A in tumor tissues than oAd/IL12/GM (***p<0.001), while the capacity of the virus to induce degradation of ECM components in tumor tissues were retained at similar level to oAd/RLX (online supplementary figure S9B). Further, oAd/IL12/GM-RLX induced a higher level of intratumoral T-cell infiltration (CD4^+^ and CD8^+^ T cells) and IFN-γ expression compared with oAd/IL12/GM (online supplementary figure S9C and S9D, ***p<0.001 or *p<0.05). Together, these results show that addition of RLX improves ECM degradation, intratumoral viral dispersion, and induction of antitumor immune response.

**Figure 4:**
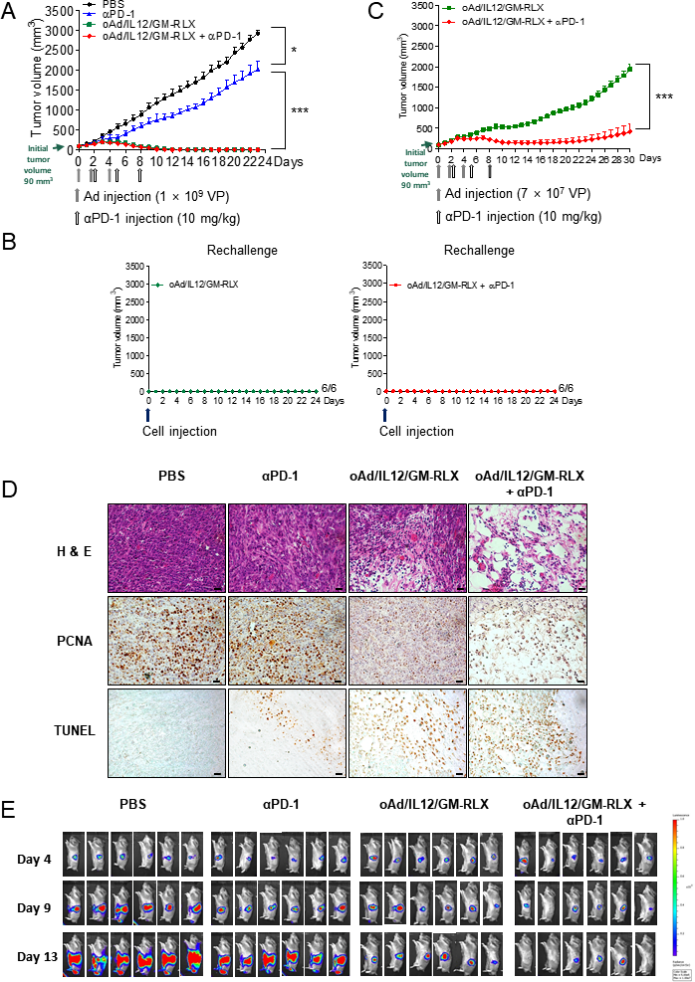
Antitumor efficacy of oAd/IL12/GM-RLX and αPD-1 combination therapy and histological and histochemical analyses. Effect of oAd/IL12/GM-RLX and αPD-1 combination therapy on tumor volume over time. Syrian hamsters were subcutaneously injected with HaP-T1 cells to establish pancreatic tumors. When the average tumor volume reached 90–100 mm^3^, (A) the tumors were injected with 1×10^9^ VP of oAd/IL12/GM-RLX (days 0, 2, and 4; gray arrows) and/or 10 mg/kg of αPD-1 (days 2, 5, and 8; empty arrows). The tumor volume was measured every day until the end of the study. Data are presented as mean±SD (n=6); PBS versus αPD-1, *p<0.05; αPD-1 versus oAd/IL12/GM-RLX or oAd/IL12/GM-RLX+αPD-1, ***p<0.001. Statistical analyses were performed by one-way analysis of variance. (B) Rechallenged tumor growth. Hamsters with complete regression (A) were rechallenged at 61 days postinoculation of the primary tumor. The PBS-treated and αPD-1-treated groups were excluded from the rechallenge experiment, as these hamsters had been euthanized due to tumor burden exceeding the ethical guidelines of our institution at the time of the rechallenge experiment. (C) The tumors were injected with 7×10^7^ VP of oAd/IL12/GM-RLX (days 0, 2, and 4; gray arrows) with or without 10 mg/kg of αPD-1 (days 2, 5, and 8; empty arrows). The tumor volume was measured every day until the end of the study. Data are presented as mean±SD (n=6); oAd/IL12/GM-RLX or oAd/IL12/GM-RLX+αPD-1, ***p<0.001. Statistical analyses were performed by two-tailed Student t-test. (D) Histological and immunohistological analysis. Tumor tissues were collected on day 10 after the first virus injection and stained with H&E, an anti-PCNA antibody, or TUNEL. Original magnification: ×400. The scale bar represents 2 µm. (E) HP-1 orthotopic pancreatic tumor-bearing hamsters were administered 1×10^9^ VP of oAd/IL12/GM-RLX into the abdomen twice (days 4 and 6), followed by a single injection into the tumor (day 8). αPD-1 was injected intraperitoneally at 3-day intervals three times (days 6, 9, and 12). The tumor volume was measured by luciferase signal at days 4, 9, and 13 after cancer cell injection. PBS, phosphate-buffered saline; αPD-1, anti-programmed cell death protein 1; Ad, adenovirus; PCNA, proliferating cell nuclear antigen; TUNEL, terminal deoxynucleotidyl transferase dUTP nick end labeling; VP, viral particle.

To evaluate the combined antitumor efficacy of oAd/IL12/GM-RLX and αPD-1, Syrian hamsters were subcutaneously injected with HaP-T1 pancreatic cancer cells. The tumor-bearing hamsters were treated with αPD-1, oAd/IL12/GM-RLX, or oAd/IL12/GM-RLX plus αPD-1, along with PBS as a negative control. As shown in figure 4A, tumors treated with PBS exhibited robust growth with the tumor volume reaching 2913±95 mm^3^ at 23 days following the initial treatment. The αPD-1-treated group showed modest tumor growth inhibition, with a 33% reduction in the average tumor volume in comparison with the PBS control group (*p<0.05). Importantly, both oAd/IL12/GM-RLX and oAd/IL12/GM-RLX plus αPD-1 treatments elicited significantly more potent antitumor effects than either PBS or αPD-1 monotherapy (***p<0.001), resulting in complete tumor regression in all six hamsters tested. To assess the establishment of adaptive immunity against the tumor re-establishment following each treatment, hamsters with complete tumor regression were rechallenged at 61 days postinoculation of the primary tumor. All of the rechallenged tumors regressed in both the oAd/IL12/GM-RLX monotherapy and αPD-1 combination treatment groups (figure 4B), demonstrating that oAd/IL12/GM-RLX was sufficient to establish durable adaptive immunity against the tumor re-establishment.

Because it was difficult to determine whether combination therapy was more effective than oncolytic Ad monotherapy when using oAd dose of 1×10^9^ VP as in figure 4A, the antitumor efficacy of oAd/IL12/GM-RLX and oAd/IL12/GM-RLX plus αPD-1 treatment was re-evaluated again at a 14.3-fold lower viral dose (7×10^7^ VP). As shown in figure 4C, oAd/IL12/GM-RLX-treated tumors continued to grow and reached 1982±126 mm^3^ by day 30 post-treatment. In marked contrast, oAd/IL12/GM-RLX plus αPD-1 treatment suppressed tumor growth, resulting in 79% tumor growth inhibition compared with the monotherapy group (***p<0.001). Importantly, 50% of the hamsters in the oAd/IL12/GM-RLX plus αPD-1 group showed complete tumor regression, whereas none of the six tumors in the monotherapy group showed complete regression. Biodistribution profiling of Ad revealed that Ad was distributed in a similar manner in oAd/IL12/GM-RLX-treated and oAd/IL12/GM-RLX plus αPD-1-treated mice, with the highest level being detected in tumor tissues (online supplementary figure S7). Likely due to the low level of Ad accumulation in normal organs following administration of either oAd/IL12/GM-RLX or oAd/IL12/GM-RLX plus αPD-1, no liver and kidney toxicities were observed, as various serum markers fell within the conventional range reported by others (online supplementary figure S8).^22–24^

An orthotopic pancreatic tumor model in Syrian hamster has been reported to closely recapitulate morphological and biological aspects of pancreatic cancer in humans.^25^ Thus, the antitumor effect of each treatment was further assessed in an orthotopic HP-1 pancreatic tumor model in Syrian hamsters. As shown in figure 4E, αPD-1 monotherapy failed to efficiently suppress orthotopic tumor growth. Orthotopic pancreatic tumors were highly aggressive and metastatic, as both PBS-treated and αPD-1-treated groups showed extensive metastasis to different sites and mortality in the subpopulation of hamsters by 11 days after the initial treatment (online supplementary figure S6A). Additionally, significant ascites accumulation, which is a prognostic marker for pancreatic cancer progression and mortality in clinic,^26^ was observed in PBS-treated or αPD-1-treated hamsters. In sharp contrast, both oAd/IL12/GM-RLX and oAd/IL12/GM-RLX plus αPD-1 induced significantly more potent primary and metastatic tumor growth inhibition, as well as complete prevention of ascites accumulation, in respect to αPD-1 monotherapy group (online supplementary figure S4E and S6). This is in line with previous report demonstrating that an oAd-mediated RLX expression has been shown to inhibit tumor metastasis,^13^ showing that localized overexpression of RLX by oAd exerts an antimetastatic effect. Collectively, these results demonstrate that oAd/IL12/GM-RLX in combination with αPD-1 can induce potent antitumor efficacy against pancreatic tumors with no observable side effects.

### Histological and immunohistochemical analyses

To further investigate the therapeutic effect of combination treatment, tumor tissues harvested from hamsters were assessed histologically and immunohistologically. As shown in figure 4D, necrotic regions were rarely detectable in tumors in the PBS-treated and αPD-1-treated groups, whereas oAd/IL12/GM-RLX plus αPD-1-treated tumors were mostly necrotic as revealed by H&E staining. Moreover, oAd/IL12/GM-RLX plus αPD-1-cotreated tumors showed a much lower density of proliferating tumor cells (PCNA-positive) than did tumors treated with monotherapy, suggesting that the combination of oncolytic Ad with αPD-1 inhibits tumor cell proliferation more effectively than monotherapy can. Furthermore, oAd/IL12/GM-RLX plus αPD-1-treated tumors contained a larger area with an apoptotic tumor cell population than did tumors treated with monotherapy, indicating that attenuated tumor cell proliferation correlated with an increased induction of apoptosis.

### Increased tumor penetration of αPD-1 after treatment with oAd/IL12/GM-RLX

**Figure 5:**
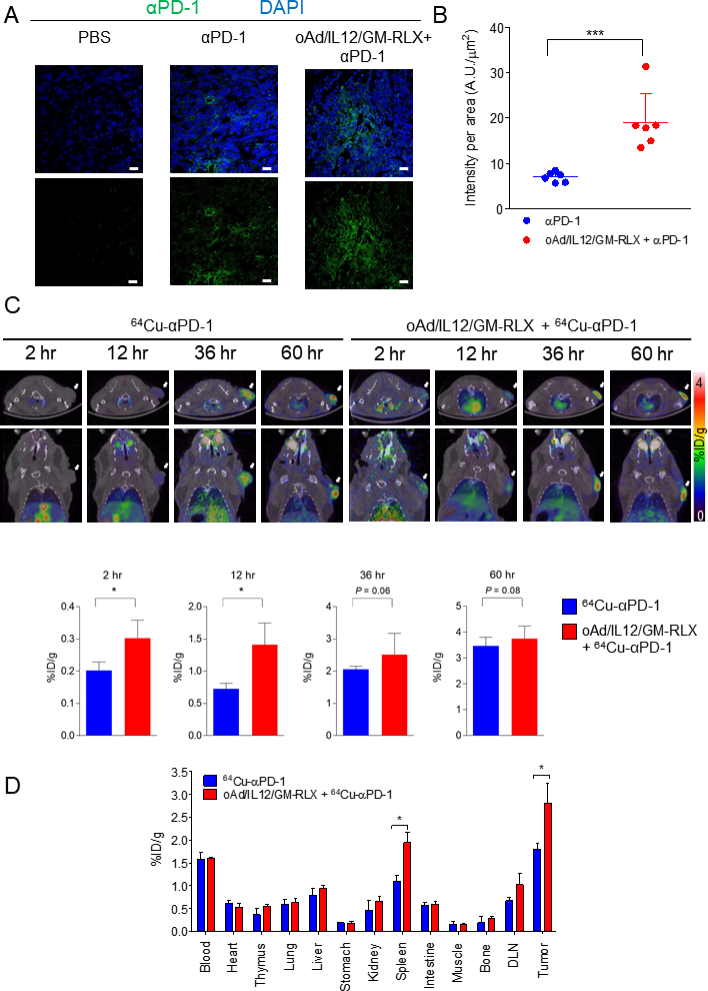
Distribution of αPD-1 Ab in HaP-T1 tumors. (A) Fluorescence images of 488-αPD-1 ab in HaP-T1 tumors. Tumors were isolated at 5 days after the final injection of PBS, 488-αPD-1, or oAd/IL12/GM-RLX plus 488-αPD-1. 488-TZB (green)-stained and DAPI-stained nuclei (blue) are shown for each group. The scale bar represents 20 µm. (B) Quantitative analysis of the accumulation of αPD-1 in tumor sections. The intensity of 488-αPD-1 and the tumor slice areas were measured in the fluorescence images. Data are presented as mean±SD (n=5); αPD-1 versus oAd/IL12/GM-RLX + αPD-1, ***p<0.001. Statistical analyses were performed by two-tailed Student t-test. (C) ImmunoPET images of subcutaneous HaP-T1 tumor-bearing hamsters. PET images were acquired for 15 min at 2, 12, and 36 hours after injection of ^64^Cu -αPD-1 AB (17.39–20.35 MBq, 400 µg of αPD-1). Tumors are indicated with the white arrows. Radioactivity in tissues are represented as %ID/g±SD (n=3); ^64^Cu-αPD-1 versus oAd/IL12/GM-RLX+^64^Cu-αPD-1, *p<0.05. Statistical analyses were performed by two-tailed Student t-test. (D) Biodistribution study after injection of ^64^Cu-αPD-1 at 60 hours. The organs and tumors were isolated from hamsters and the radioactivity was measured. Radioactivity in tissues is represented as %ID/g±SD (n=3); ^64^Cu-αPD-1 versus oAd/IL12/GM-RLX+^64^Cu-αPD-1, *p<0.05. Statistical analyses were performed by two-tailed Student t-test. αPD-1, anti-programmed cell death protein 1; Ab, antibody; DAPI, 4,6-diamidino-2-phenyindole; PBS, phosphate-buffered saline; DLN, draining lymph node; PET, positron emission tomography.

The distribution of a therapeutic Ab, TZB, in tumor tissue was enhanced by oncolytic Ad-mediated intratumoral expression of RLX (figures 1–3). On the basis of these results, we hypothesized that a combination of oAd/IL12/GM-RLX and αPD-1 might elicit synergistic antitumor effects because RLX would facilitate the intratumoral penetration of αPD-1. To investigate whether oAd/IL12/GM-RLX increased the intratumoral penetration of αPD-1 into desmoplastic pancreatic tumor tissues, oAd/IL12/GM-RLX alone, Alexa 488-conjugated αPD-1 alone (488-αPD-1), or a combination of these treatments was administered. αPD-1As shown in figure 5A, B, the amount of αPD-1 penetration in tumor tissue increased 2.7-fold in the combination therapy group compared with αPD-1 monotherapy group at 7 days of postinitial treatment (***p<0.001), demonstrating that oAd/IL12/GM-RLX enhances the penetration of αPD-1 into tumor.

To further evaluate the tumor uptake of αPD-1 and its whole-body distribution, we acquired immunoPET images of HaP-T1 tumor bearing hamsters injected with ^64^Cu-conjugated αPD-1 (^64^Cu-αPD-1) with/without oAd/IL12/GM-RLX in the same regimen as described earlier (figure 5C). The intratumoral uptake of ^64^Cu-αPD-1 in the combination therapy group was higher than that in the ^64^Cu-αPD-1 monotherapy group at all time points. The biodistribution of ^64^Cu-αPD-1 was calculated as %ID/g in various organs and tumor tissues at 60 hours of postinjection (figure 5D). The uptake of ^64^Cu-αPD-1 to tumor tissues or spleen was significantly higher in the combination therapy group than in the monotherapy group (1.56-fold or 0.84-fold higher in respective tissues, *p<0.05). In addition, the volume of DLN, a T cell-enriched site, increased by 48-fold following oAd/IL12/GM-RLX treatment (online supplementary figure S5). Taken together, these results suggest that a combination of oAd/IL12/GM-RLX and αPD-1 enhances T-cell expansion and activation as well as augments αPD-1 colocalization in T cell-enriched tissues to promote robust antitumor immunity.

### Increased cytotoxic T-cell activation and infiltration into tumor induced by coexpression of IL-12, GM-CSF, and RLX

**Figure 6:**
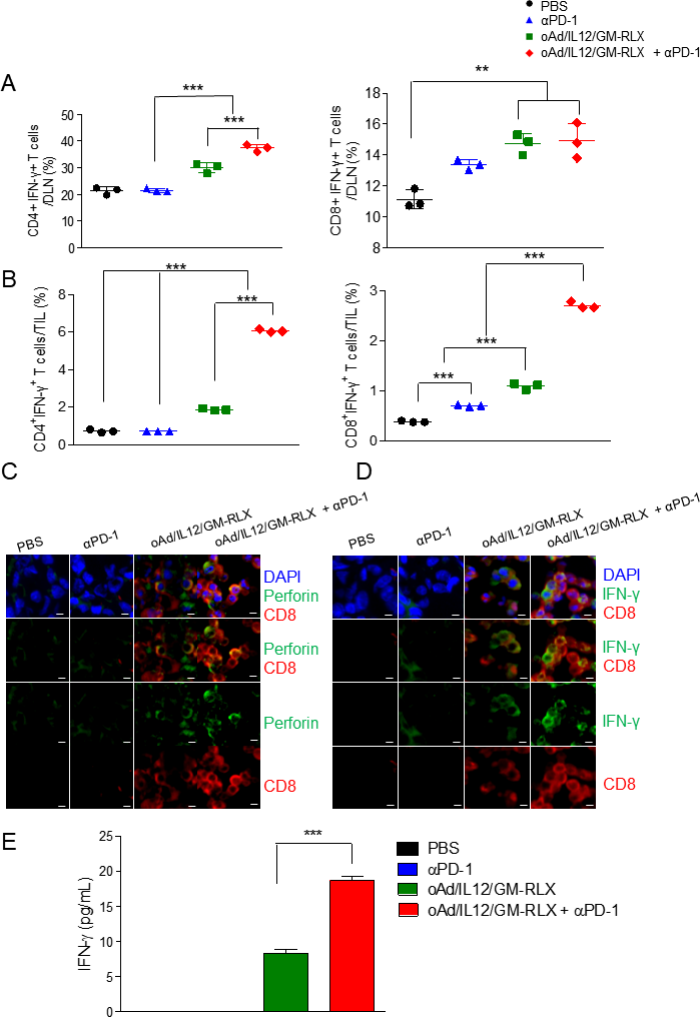
Analysis of activated T cells in DLN and tumor tissue. HaP-T1 tumor-bearing hamsters were injected with PBS, αPD-1 (10 mg/kg), oAd/IL12/GM-RLX (7×10^7^ VP), or oAd/IL12/GM-RLX (7×10^7^ VP) plus αPD-1 (10 mg/kg). Lymphocytes were harvested 12 days after the initial treatment to perform FACS analysis for CD4^+^IFN-γ^+^ or CD8^+^ IFN-γ^+^ T cells in (A) DLN or (B) tumor tissue. Data are presented as mean±SD of three independent measurements; **p<0.01, ***p<0.001. (C, D) Double-immunofluorescence staining for colocalization of CD8 (red) and perforin or IFN-γ (green) in tumor tissues treated with PBS, oAd/IL12/GM-RLX, αPD-1, or oAd/IL12/GM-RLX plus αPD-1 and isolated at 5 days after the ﬁnal treatment. The scale bar represents 5 µm. (E) Lymphocytes were collected from hamster DLNs at day 12 after the initial virus injection and coincubated with preirradiated HaP-T1 cells. After 3 days of coincubation, an IFN-γ ELISA was carried out. Data are presented as mean±SD (n=3); oAd/IL12/GM-RLX versus oAd/IL12/GM-RLX+αPD-1, ***p<0.001. Statistical analyses were performed by one-way analysis of variance. PBS, phosphate-buffered saline; αPD-1, anti-programmed cell death protein 1; DAPI, 4,6-diamidino-2-phenyindole; DLN, draining lymph node; FACS, fluorescence-activated cell sorting; IFN, interferon.

To explore whether increased penetration of αPD-1 into tumor induces the infiltration and activation of T cells, the accumulation of IFN-γ-expressing CD4^+^ or CD8^+^ T cells was assessed in the DLN, which functions as an initial site of expansion for cytotoxic T cells. The number of IFN-γ-expressing CD8^+^ T cells in DLN of oAd/IL12/GM-RLX plus αPD-1 and oAd/IL12/GM-RLX groups were similarly high, while the number of IFN-γ-expressing CD4^+^ T cells was highest in the DLN of oAd/IL12/GM-RLX plus αPD-1 group (figure 6A, ***p<0.001), suggesting a greater number of activated antitumor T cells are present in the lymphoid organ following combination therapy. Even if large quantities of activated T cells are present in various immune organs such as the DLN, antitumor immune responses may still be inadequate for clinical benefit because of inefficient intratumoral infiltration of these activated T cells.^27^ Thus, the intratumoral infiltration of activated T cells (CD4^+^IFN-γ^+^ T cell and CD8^+^IFN-γ^+^ T cell subsets of the tumor-infiltrating lymphocytes (TILs)) was evaluated by flow cytometry. As shown in figure 6B, both CD4^+^IFN-γ^+^ T cells and CD8^+^IFN-γ^+^ T cells accumulated at a higher frequency in combination therapy-treated tumors than in the αPD-1 or oAd/IL12/GM-RLX monotherapy groups (***p<0.001), suggesting that concomitant administration of immunostimulatory oncolytic Ad and αPD-1 can lead to enhanced T cell-mediated antitumor immune responses. These results were also confirmed by immunofluorescence staining of tumor tissues treated with PBS, αPD-1, or oAd/IL12/GM-RLX plus αPD-1. As shown in figure 6C, D, both CD8^+^IFN-γ^+^ and CD8^+^perforin^+^ T cells were more frequently detected in oAd/IL12/GM-RLX plus αPD-1-treated tumors compared with tumors from the monotherapy groups. Taken together, these results suggest that the combined administration of oAd/IL12/GM-RLX and αPD-1 resulted in the attraction of more activated T cells and facilitated their infiltration into poorly immunogenic tumors.

To assess cancer-specific immune responses, DLN-based immune cells from hamsters treated with αPD-1, oAd/IL12/GM-RLX, or oAd/IL12/GM-RLX plus αPD-1 were harvested and cocultured with irradiated HaP-T1 cancer cells. After 3 days, the supernatant was harvested, and the levels of IFN-γ secreted by cancer-specific lymphocytes were measured by ELISA. As shown in figure 6E, IFN-γ expression was not detected in the αPD-1-treated group, suggesting that ICI alone cannot sufficiently induce antitumor immune responses in poorly immunogenic tumors. In marked contrast, both oAd/IL12/GM-RLX monotherapy and combination therapy with ICI resulted in a markedly elevated IFN-γ expression level, which was 2.2-fold higher in the combination therapy group versus the monotherapy group (***p<0.001). Together, these results suggest that immune stimulatory oncolytic Ad can induce tumor-specific immune responses in ‘cold’ tumors and that this effect can further be enhanced by concomitant ICI administration.

### Overcoming the ICI resistance of poorly immunogenic tumors with oAd/IL12/GM-RLX

**Figure 7:**
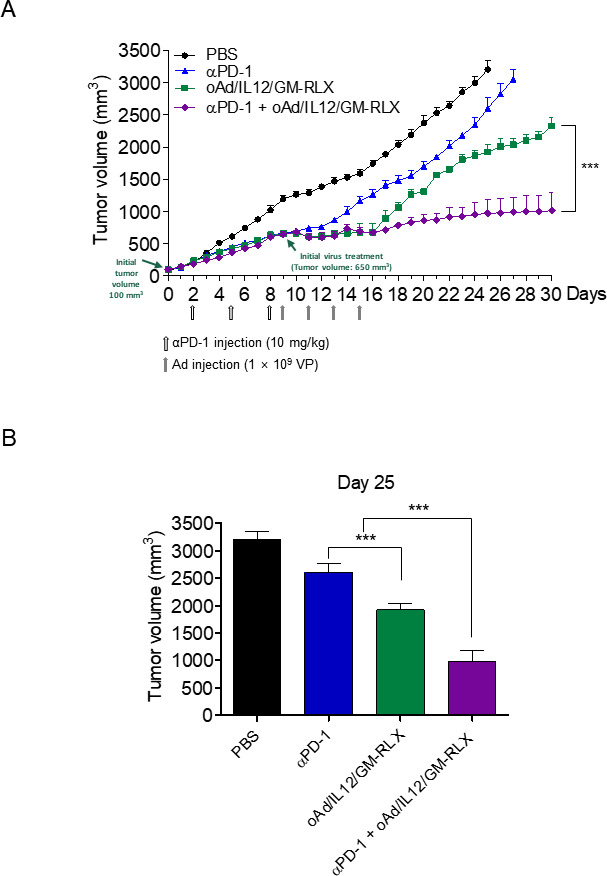
Overcoming αPD-1 resistance with oAd/IL12/GM-RLX. (A) Syrian hamsters were subcutaneously injected with HaP-T1 cells to establish pancreatic tumors. When the average tumor volume reached 90–100 mm^3^, the hamsters were injected with 10 mg/kg of αPD-1 intraperitoneally (days 2, 5, and 8; empty arrows) and 1×10^9^ VP of oAd/IL12/GM-RLX intratumorally (days 9, 11, 13, and 15; gray arrows). The tumor volume was measured every day until the end of the study. Data are presented as mean±SD (n=5); oAd/IL12/GM-RLX versus αPD-1+oAd/IL12/GM-RLX, ***p<0.001. Statistical analyses were performed by one-way ANOVA. (B) Bar graph of the tumor size at day 25. The data were based on (A). Data are presented as mean±SD (n=5); oAd/IL12/GM-RLX versus αPD-1 or αPD-1+oAd/IL12/GM-RLX, ***p<0.001. Statistical analyses were performed by one-way ANOVA. PBS, phosphate-buffered saline; αPD-1, anti-programmed cell death protein 1; Ad, adenovirus; ANOVA, analysis of variance; VP, viral particle.

A large subset of patients receives no clinical benefits from ICIs and tumor hyperprogression has been reported in some cases.^28^ To explore whether subsequent administration of oAd/IL12/GM-RLX provides therapeutic benefits in such ICI refractory subsets, the inhibitory effect of oAd/IL12/GM-RLX on tumor growth was assessed in tumors pretreated with ICI therapy. oAd/IL12/GM-RLX was administered four times at 2-day intervals when tumor growth was no longer inhibited by αPD-1 monotherapy (9 days postinitial treatment as determined in figure 4A). As shown in figure 7A, αPD-1 as monotherapy failed to suppress the tumor growth, although the tumor volume was steady for about 4 days after administration (tumor growth rates: 29.4 mm^3^/day during days 8–12 vs 144.2 mm^3^/day during days 12–28). In sharp contrast, the administration of oAd/IL12/GM-RLX following αPD-1 treatment continued to suppress tumor growth during both early and late time intervals (tumor growth rates: 9.8 mm^3^/day during days 9–17 vs 22.6 mm^3^/day during days 17–30) more effectively compared with αPD-1 monotherapy, suggesting that oncolytic Ad can effectively treat ICI refractory tumors of high tumor burden. On day 25 postadministration, oAd/IL12/GM-RLX in αPD-1 resistant tumor (αPD-1+oAd/IL12/GM-RLX) still exhibited most potent tumor growth inhibition when compared with PBS and αPD-1 treatment, showing that oAd/IL12/GM-RLX can suppress tumor growth effectively in hamsters with ICI refractory tumors of high tumor burden at the time of virus injection: specifically, αPD-1 Ab treatment followed by oAd/IL12/GM-RLX yielded a 62.5% and 49.3% lower tumor burden than did αPD-1 or oAd/IL12/GM-RLX treatment alone, respectively (figure 7B, ***p<0.001). Taken together, these results suggest that administration of oAd/IL12/GM-RLX to ICI refractory tumors could induce durable tumor growth suppression and be a beneficial therapeutic regimen for patients who are resistant to ICI monotherapy.

## Discussion

A hostile tumor microenvironment remains a critical challenge to achieving a high therapeutic index with cancer therapies. Several factors in the tumor environment, such as dense ECM, poor vascular permeability, hypoxia, and poor lymphatic drainage, restrict intratumoral infiltration and dispersion of drugs and therapeutic cells.^3 29^ Recently, there was a report that the efficacy of immune check point blockade could be enhanced using intratumorally injected GM-CSF armed oncolytic Ad.^30^ However, in the solid tumor, overcoming physical barrier as well as physiological barrier would be important. Previously, we showed RLX could degrade the ECM within the tumor.^13 20^ Similarly, RLX conjugated superparamagnetic iron oxide nanoparticles showed the degradation of fibrosis in pancreatic cancer, inducing enhancement of efficacy of gemcitabine.^31^

In our present study, to overcome the physical barrier of solid tumor, oncolytic Ad expressing RLX was used in combination with therapeutic Abs (TZB and αPD-1) and radiolabeled Abs (^64^Cu-TZB and ^64^Cu-αPD-1) in the present report to promote drug penetration through degradation of the tumor ECM. Moreover, to overcome the immunological barrier of solid tumor, we used ‘armed oncolytic Ad’ (oAd/IL12/GM-RLX) for the treatment of pancreatic tumors that are refractory to ICI. Of note, oAd/IL12/GM-RLX coexpresses genes encoding four proteins, IL-12 consisting of p35 and p40 subunits, GM-CSF and RLX. This is the first report demonstrating that four genes (IL-12p35, IL-12p40, GM-CSF, and RLX) can be effectively transferred by a single viral vector. oAd/IL12/GM-RLX showed a more potent antitumor effect than did previously reported oAds at a dose that is at least 10-fold lower.^32^ Furthermore, oAd/IL12/GM-RLX remodeled the physical and immunological barriers in poorly immunogenic solid tumors, leading to enhanced potency of ICI.

TZB is a monoclonal Ab specific to human epidermal growth factor receptor 2 (Her2) that induces its therapeutic effects in Her2-positive cancer by directing Ab-dependent cell-mediated cytotoxicity.^33^ However, the therapeutic efficacy of such Abs is limited because of insufficient penetration into solid tumors. Only 0.001%–0.01% of the injected therapeutic Abs reach the tumor of patients.^34 35^ To overcome this limitation, researchers have manipulated the size, charge, and antigen-binding affinity of Abs to increase their penetration and dispersion.^36^ Alternatively, therapeutic Abs have been coadministered with other substances to increase vascular permeability or reduce epithelial barriers.^37 38^ In the present study, we used an RLX-expressing oncolytic Ad to increase intratumoral Ab accumulation through degradation of the ECM.

Indeed, oAd/RLX was able to effectively degrade aberrant tumor ECM (online supplementary figure S1C), thus resulting in significantly enhanced penetration and dispersion of TZB (figure 2A–C). Of note, TZB penetrated significantly more into the center of the tumor tissue when coadministered with oAd/RLX, even though the vascular density was not altered (online supplementary figure S3). In addition, ECM reduction by RLX increased the sensitivity of PET imaging by enhancing tumor uptake of radiolabeled Abs (figure 3). These results were likely achieved by attenuation of interstitial pressure following epithelial cell junction opening, RLX-mediated degradation of the ECM, and oncolysis of tumor, ultimately relieving mechanical constraints on the tumor tissue and creating more interstitial space for drugs to maneuver.^39^ Furthermore, oncolytic Ad-mediated expression of RLX has been reported to promote apoptotic tumor cell death,^13^ which in theory could also lower the interstitial pressure in solid tumors.^40^ Our strategies enhancing tumor uptake of radiolabeled-Abs are expected to be also useful for α-radioimmunotherapy if ^64^Cu is changed to α-particle, such as ^211^At (t_1/2_, 7.2 hours) and ^213^Bi (t_1/2_, 46 min).

Among various cancer-targeted therapeutic Abs used in the clinic, ICIs are unique in their immune modulatory ability, which allows the host immune system to overcome tumor-induced immunosuppression and immune escape mechanisms to mount a systemic antitumor immune response against cancer, yielding remarkable achievements in clinical trials involving patients with cancers originating from various tissues.^41^ However, ICI monotherapy elicits insufficient antitumor efficacy in some patients due to various immune escape mechanisms of tumors.^42^ Particularly, the tumor microenvironment functions as a limiting factor for ICIs, as it does for other chemotherapeutics for which penetration of immune cells is hindered by physical barriers such as tight epithelial junctions and a dense layer of tumor ECM. ‘Cold’ tumors, which are refractory to ICIs, often possess a dense ECM, resulting in poor penetration of activated immune cells in a large subset of patients,^43^ with only 20%–30% benefiting from ICI therapy.^44^ Among cold tumors, pancreatic cancer is particularly problematic due to a strong desmoplastic reaction leading to the formation of dense tumor ECM.^45^

On the basis of these reports, we hypothesized that an RLX-expressing oncolytic Ad may enhance the efficacy of ICIs through ECM degradation. Indeed, the degradation of tumor ECM induced by oAd/IL12/GM-RLX led to enhanced ICI penetration, similar to what was observed with the combination of oAd/RLX and TZB as shown in figures 1–3 and efficient infiltration of activated T cells in highly desmoplastic pancreatic tumor tissues (figures 5 and 6). These attributes are highly favorable for the treatment of pancreatic cancer, as patients with pancreatic cancer with high density and quantity of TIL have been shown to exhibit higher overall survival rate and progression-free survival than those with low TIL count.^46^ In line with our findings, a phase II clinical trial, which combines IL-12-expressing plasmid DNA and αPD-1 Ab, demonstrated that IL-12 expression enhances the response to αPD-1 therapy in patients with low levels of TILs.^47^ Because viral vectors, especially Ad, often express therapeutic genes at a much higher level than plasmid vectors (the difference is further magnified when oncolytic vectors are compared due to the exponential amplification in therapeutic gene expression following viral replication), immune stimulatory oncolytic viral vectors in combination with ICIs may yield promising results against cold tumors in future clinical trials.

Another major limitation of ICI therapy is disease relapse, and recurrence among responding patients as drug-induced selective pressure leads to the survival of tumor cells with adaptive resistance against ICIs. Tumor cells frequently escape ICI-mediated immune modulation by upregulation of an alternative immune checkpoint.^48^ Currently, various coadministration strategies are being evaluated and have demonstrated promising results in clinical trials.^49^ In line with these trends, combined administration of an ICI and oAd/IL12/GM-RLX for the treatment of pancreatic tumors that are refractory to ICI led to durable and potent tumor growth suppression, whereas either immunotherapeutic alone eventually resulted in tumor progression (figure 7), demonstrating that oAd/IL12/GM-RLX could convert ICI refractory cold tumors to ‘hot’ tumors, thus overcoming the poor efficacy of ICI monotherapy against non-immunogenic tumors.

Our initial assessment of combination therapy using TBZ and oAd/RLX via systemic administration was performed in a human tumor xenograft model using immunodeficient nude mice, focusing on whether RLX degrades dense tumor ECM originating from human cancer. Following our initial assessment clearly illustrating that oAd/RLX-mediated degradation of tumor ECM can improve TBZ penetration and accumulation in tumor tissues, we hypothesized that similar enhancement of efficacy can be achieved by combining RLX-expressing oAd with other Ab therapeutics. To evaluate our second assertion, immunocompetent subcutaneous or orthotopic pancreatic tumor models in Syrian hamsters were used to ascertain that oAd/IL12/GM-RLX in combination with αPD-1 enhances ICI distribution in tumor tissues, as well as promotes intratumoral infiltration and activation of T cells. An orthotopic pancreatic tumor model in Syrian hamster was reported to closely recapitulate morphological and biological aspects of pancreatic cancer in human.^25^ For the study in an immune-competent host, intratumoral injection was used due to the technical hurdles toward intravenous administration in hamsters where tail vein is unavailable. Use of femoral vein was also not suitable for long-term follow-up (~30 days). Despite intravenous injection being preferred route of injection over local administration methods, intratumoral or intralesional injection methods were still widely used in clinics.^50^

In sum, an RLX-expressing oncolytic Ad can greatly enhance Ab penetration and distribution by degrading and remodeling the tumor ECM, demonstrated in both subcutaneous and orthotropic tumor model, leading to potent combination therapy effect against both primary and metastatic tumors. Further, concomitant expression of two immune stimulatory cytokines (IL-12 and GM-CSF) and RLX by a single oncolytic Ad vector led to a significant improvement in tumor growth inhibition. Prominently, a combination of oAd/IL12/GM-RLX and αPD-1 Ab improved the accumulation of activated T cells in desmoplastic tumors, which was achieved by increasing the intratumoral penetration of the Ab, antitumor cytokine expression, and ECM degradation. Collectively, a combination of oncolytic Ad coexpressing immune stimulatory and ECM degrading factors with αPD-1 induced durable tumor growth suppression of ICI refractory cold tumors in high tumor burden scenarios by favorably modulating both physical and immunological aspects of poorly immunogenic solid tumor.
