# Supplementary material for: Relaxin-expressing oncolytic adenovirus induces remodeling of physical and immunological aspects of cold tumor to potentiate PD-1 blockade
Source: J Immunother Cancer. 2020 Aug 4;8(2):e000763. doi: 10.1136/jitc-2020-000763 (PMC7406118; doi:10.1136/jitc-2020-000763)
Supplement: Supplementary data [file jitc-2020-000763supp001.pdf]

Submitted Manuscript: Confidential

Relaxin-expressing oncolytic adenovirus induces remodeling of physical and immunological aspects of cold tumor to potentiate PD-1 blockade

Bo-Kyeong Jung[1†], Hae Young Ko[2†], Hyun-ji Kang[2], JinWoo Hong[1,3], Hyo Min Ahn[1,3], Youjin Na[1], Hyeon-gi Kim[2], Jin Su Kim[2,4*], Chae-Ok Yun[1,3,5*]

[1]Department of Bioengineering, College of Engineering, Hanyang University, 222 Wangsimni-ro, Seongdong-gu, Seoul 04763, Republic of Korea

[2]Division of Radiation Biomedical Research, Korea Institute of Radiological and Medical Sciences, 75 Nowon-ro, Nowon-gu, Seoul 01812, Republic of Korea

[3]GeneMedicine Co., Ltd., Fusion Technology Center (FTC), 222 Wangsimni-ro, Seongdong-gu, Seoul, 04763, Republic of Korea

[4]Radiologcial and Medico-Oncological Sciences, University of science and technology (UST), 75 Nowon-ro, Nowon-gu, Seoul 01812, Republic of Korea

[5]Institute of Nano Science and Technology (INST), Hanyang University, 222 Wangsimni-ro, Seongdong-gu, Seoul 04763, Republic of Korea

*To whom correspondence should be addressed: Chae-Ok Yun; chaeok@hanyang.ac.kr and Jin Su Kim; kjs@kirams.re.kr.

†These authors contributed equally to this work.

Supplemental Materials & Methods

RT-PCR analysis

Total RNAs were extracted from Ad-infected NCI-N87 or HaP-T1 cell lysates using an RNA iso Plus kit (Takara, Otsu, Japan) according to the manufacturer's protocol. cDNA was prepared from 1 μg of total RNA with a High Capacity cDNA Reverse Transcription Kit (Applied Biosystems, Foster City, CA) under the following incubation conditions: 25°C for 10 min, 37°C for 120 min, and 85°C for 5 min. The RLX sequence was amplified by PCR with the following primer set: 5'-CCTGGAGCAAAAGGTCTCTG-3' as the sense primer and 5'-TCTCAGATAGGGCTGCCTTC-3' as the antisense primer.

Masson's trichrome staining and Safranin-O staining

To investigate the expression of collagen in tumor tissues, Masson's trichrome (MT) staining was performed on sectioned tumor tissues using a MT staining kit (DAKO, Glostrup, Denmark) according to the manufacturer's protocol. To further investigate the side effects of oAd/RLX, oAd/RLX (2.5×10^7 VP) was administered intravenously on day 0, 2, and 4 to BALB/c nude mice. At 5 days after the first injection of virus, the articular cartilage in the knee joint was isolated from mice and the proteoglycan content assessed by Safranin-O staining according to the manufacturer's protocol.

IL-12 and GM-CSF ELISA

HaP-T1 cells were plated onto 6-well plates at 2×10^5 cells per well, and then infected with oAd/IL12/GM-RLX at 0.2-5 MOI. At 48 hr post infection, the expression levels of IL-12 and GM-CSF were quantified in culture supernatants using a mouse IL-12 p70 DuoSet ELISA

kit (R&D Systems, Minneapolis, MN) and a mouse GM-CSF DuoSet ELISA kit (R&D Systems), respectively, according to the manufacturer's recommendations. Serial dilutions of a known concentration of purified recombinant mouse IL-12 or GM-CSF were used to establish a standard curve.

Orthotopic pancreatic tumor model

To establish the hamster orthotopic pancreatic tumor model, luciferase-expressing HP-1 hamster pancreatic cancer cells (4×10^5) were injected directly into the tail of pancreas, and the establishment of the orthotopic pancreatic tumor model was confirmed by bioluminescence imaging. Hamsters were anesthetized in a chamber filled with 2% isoflurane in O_2 and received D-luciferin (150 mg/kg; Caliper, Hopkinton, MA) by intraperitoneal injection. Then, both photographic and luminescent images were attained from the anesthetized hamsters using the IVIS imaging system (Xenogen, Alameda, CA). When tumor establishment was confirmed, hamsters were randomized into four groups. The 1×10^9 VP of oAd/IL12/GM-RLX was injected into the abdomen twice (day 4 and 6), followed by a single injection into the tumor (day 8). αPD-1 was injected intraperitoneally at 3-day intervals for 3 times (day 6, 9, 12). After 11 days after initial virus injection, the ascites was harvested using 10 mL syringe and luciferase images were obtained from various organ (heart, lung, liver, kidney, spleen, intestine, seminal vesicle, and prostate) and tumor to assess the therapeutic effect and metastasis.

Biodistribution

When the HaP-T1 subcutaneous tumor volume had reach approximately 120-150 mm^3, tumor-bearing hamsters were injected intratumorally with 7×10^7 VP of oAd/IL12/GM-RLX

three times (day 0, 2, 4) with/without intraperitoneally administered 10 mg/kg of αPD-1 three times (day 2, 5, 8), along with PBS as control. The liver, spleen, kidney, lung, heart, and tumor were harvested at 7- or 14-day post initial virus treatment, and genomic DNA was extracted from the tissues using the QIAamp DNA Mini Kit (Qiagen, Hilden, Germany) according to the manufacturer's recommendation. The copy number of viral genomes in each sample was measured by real-time quantitative PCR (Applied Biosystems, Forster City, CA) as previously reported [1].

In vivo *toxicity*

To assess potential *in vivo* toxicity of each treatment group, hamsters were injected intratumorally with 7×10^7 VP of oAd/IL12/GM-RLX three times (day 0, 2, 4) and/or intraperitoneally 10 mg/kg of αPD-1 three times (day 2, 5, 8), along with PBS as control. The serum levels of blood urea nitrogen (BUN), creatinine, aspartate aminotransferase (AST), and alanine transaminase (ALT) was measured at 3 days after last treatment.

Dosimetry

To assess the safety of ^{64}Cu-TZB or oAd/RLX plus ^{64}Cu-TZB, the effective doses in normal organs were calculated. To assess the therapeutic efficacy of ^{64}Cu-TZB or oAd/RLX plus ^{64}Cu-TZB, the absorbed doses in tumor regions were calculated. The radiation dose per unit of administered activity (mSv/MBq), the effective dose in organs, and the absorbed dose for the tumor region on PET scans in the mouse were calculated using OLINDA/EXM software (OLINDA; Vanderbilt University, Nashville, TN). For the calculation of absorbed dose in tumors, the OLINDA sphere model was used. The tumor volume was calculated on PET data

with multiple slices of ROIs. The tumor mass was calculated with the assumption of 1 g/mL. X-ray CT data were used for the delineation of ROIs. ROIs were delineated in the brain, lung, liver, stomach, intestine, kidney, and tumor regions. The size of ROIs ranged from 0.0067 to 0.066 cm^2. After delineation of ROIs on X-ray CT scans, ROIs were copied to the ^{64}Cu-TZB PET data.

Time activity curves (TACs) were obtained for each organ. Decay-uncorrected TACs were derived and cumulative activity was obtained from the AUC for TACs. For each source organ, the residence time was calculated by dividing the cumulative activity by the total injected dose.

The absorbed S value for each tumor volume was calculated with scaling by mass. A non-linear fitting between the S value and the mass was used because linear interpolation could lead to the value of S being too large.

Antitumor effect comparison of oncolytic adenoviruses expressing one, two, or three of RLX, IL-12, and/or GM-CSF

An oncolytic Ad expressing RLX (oAd/RLX), IL-12 and GM-CSF-expressing oncolytic Ad (oAd/IL12/GM) and IL-12, GM-CSF, and RLX-expressing oncolytic Ad (oAd/IL12/GM-RLX) was used along with oAd control. The generation of oAd and oAd/IL12/GM has been previously reported [2]. For oAd/RLX constructs, the E3 shuttle vector containing the RLX expression cassette [3, 4] was incorporated to the modified Ad5 total vector by homologous recombination as previously described [5, 6]. The construction method of oAd/IL12/GM-RLX was described in Material & Method section of this manuscript. Pancreatic tumors were established subcutaneously on the right flank of Syrian golden hamsters by inoculating 3×10^6 HaP-T1 cells suspended in 50 μL of Hank's balanced salt solution (Gibco-BRL, Grand Island,

NY). When the average tumor volume reached 100 mm^3, 1 × 10^8 VP of each virus was intratumorally administered to the tumor-bearing hamsters at day 0, 2, and 4. All treatments began when the average tumor volume was approximately 100 mm^3. Tumor growth was evaluated every day by taking measurements of the L and W of the tumor. Tumor volume was calculated using the following formula: volume = $0.523L(W)^2$.

Quantification of IL-12, GM-CSF, and IFN-γ in tumor tissues

The tumor tissues were lysed in NP-40 buffer (ELPIS Biotech, Daejeon, Korea) with a proteinase inhibitor cocktail (Sigma, St. Louis, Mo). Homogenates were then centrifuged, and the supernatants were harvested. Total protein quantities were determined using a bicinchoninic acid protein assay reagent kit (Thermo Fisher Scientific, Waltham, MA). Levels of IL-12 (G-Biosciences, St Louis, MO), GM-CSF (Mybiosource, San Diego, CA), and IFN-γ (Mybiosource) in the tumor tissue extract were determined by ELISA according to instructions from the manufacturer. ELISA data were normalized relative to the total protein concentration in each tumor and were presented as picograms per milligram of total protein.

Immunohistochemical analysis

For immunohistochemical analysis, tumor tissues were collected from hamsters at 7 days post initial Ad injection, embedded in paraffin, and sectioned at 4 μm thickness. The tumor sections were blocked with Blocking Solution (DAKO) for 2 hr. The tumor sections were then incubated with a mouse anti-collagen type I Ab (Cell Signaling Technology, Beverly, MA), anti-collagen type III Ab (Sigma), or anti-E1A Ab (Santa Cruz biotechnology, Santa Cruz, CA) as primary Ab. After washing, the sections were incubated with the secondary Ab matching each

and then counterstained with Meyer's hematoxylin (Sigma). To identify lymphocyte infiltration into tumor tissues, tumor tissues from hamsters were frozen in OCT compound (Sakura Finetec, Torrance, CA) and cut into 5 μm sections. Tumor sections were fixed with chilled acetone for 10 min and blocked with Blocking Solution (DAKO). Sections were then incubated with primary Abs, mouse anti-rat CD4 monoclonal Ab (ebioscience, San Diego, CA) and mouse anti-rat CD8 monoclonal Ab (ebioscience), at 4°C overnight. After washing three times with PBS, samples were incubated with the secondary Ab, goat anti-mouse IgG(H+L)-HRP (Southern biotech, Birmingham, AL) at room temperature for 2 hr. In the final step, the slides were washed with PBS, then counterstained with Meyer's hematoxylin (Sigma). The image was analyzed under a fluorescence microscope. The positive staining region was quantified with MetaMorph software.

Graphical Abstract

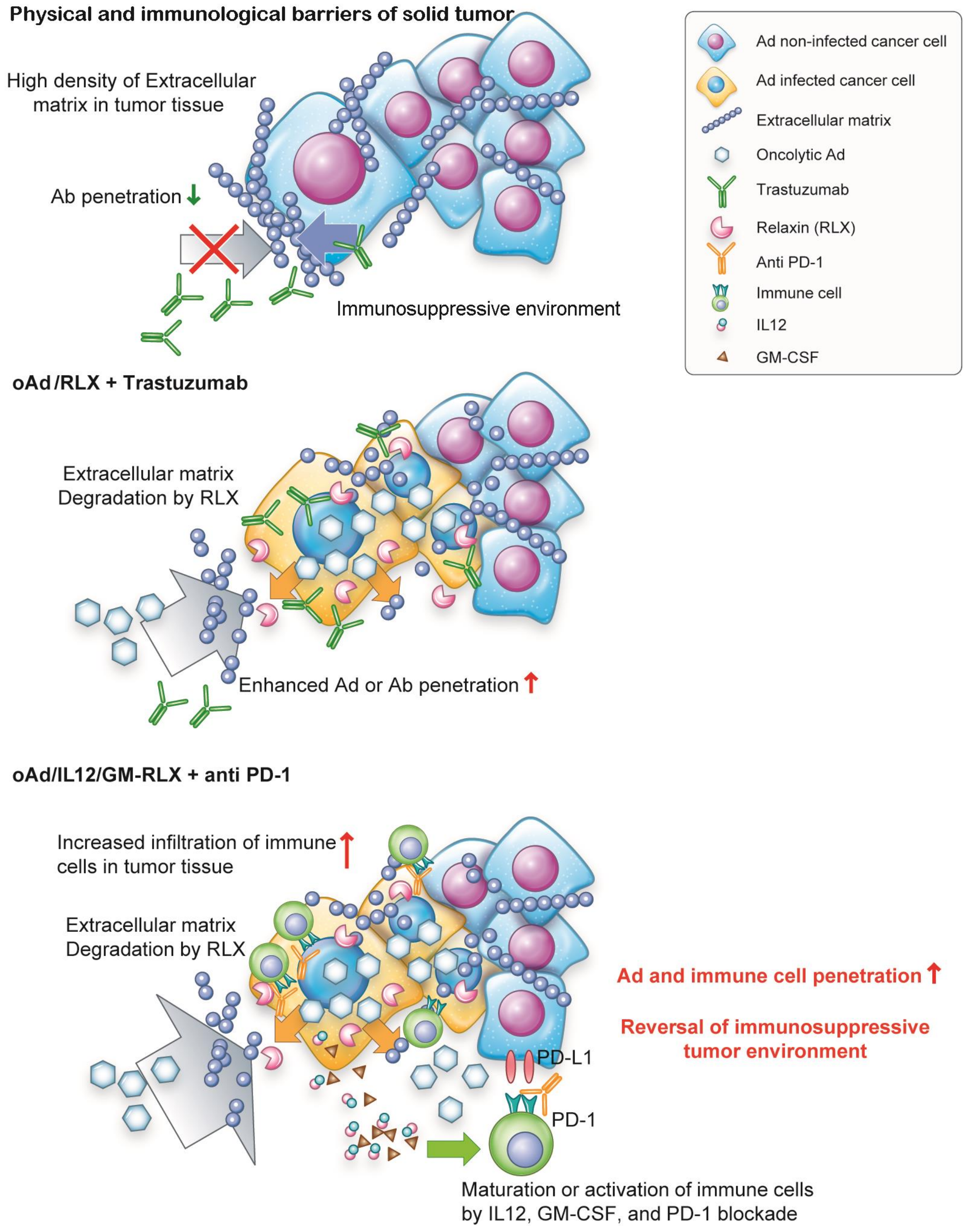

Supplementary Figure 1

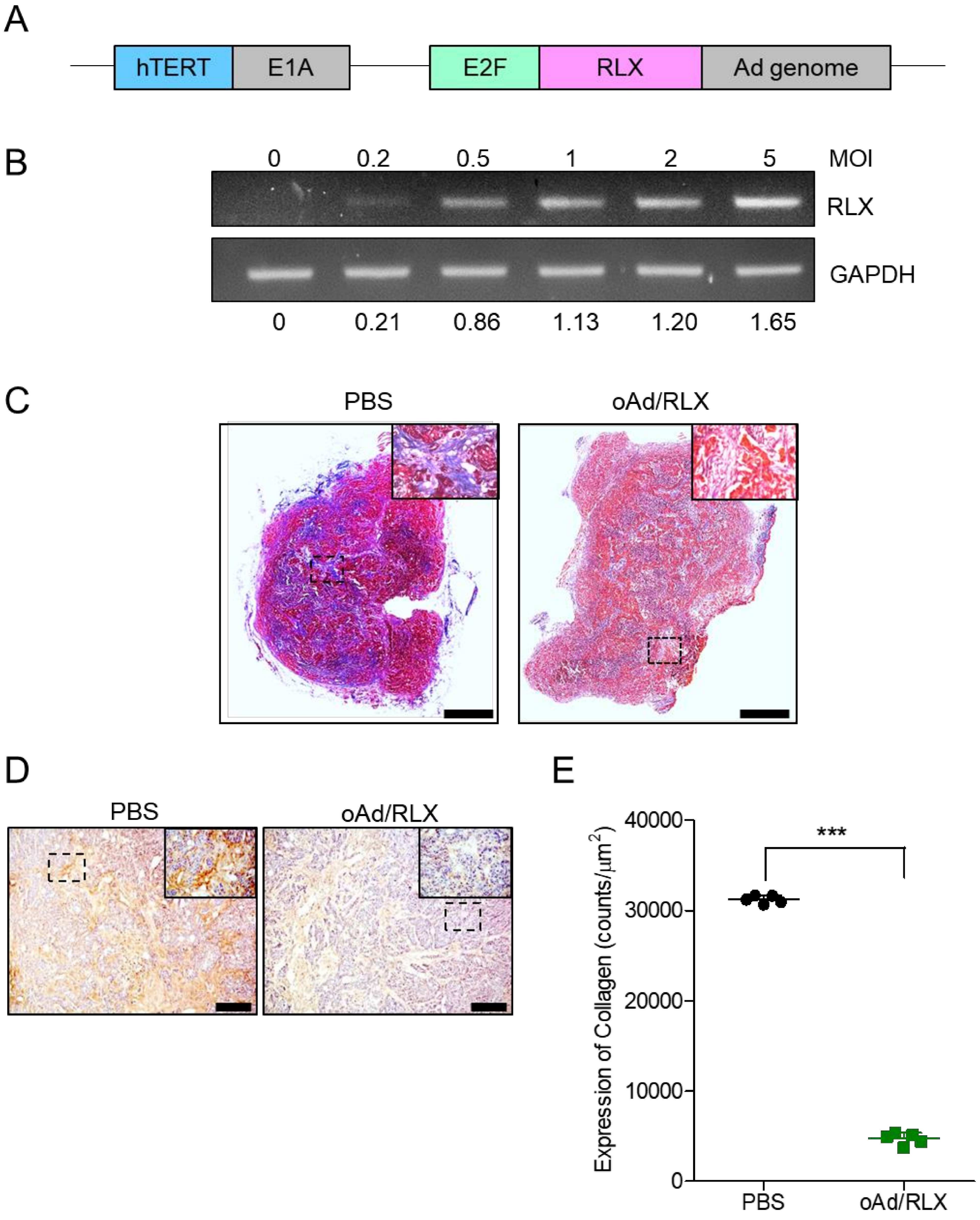

1

2

BMJ Publishing Group Limited (BMJ) disclaims all liability and responsibility arising from any reliance placed on this supplemental material which has been supplied by the author(s)

Fig. S1. Characterization of oAd/RLX. (A) Genome structure of oAd/RLX. (B) oAd/RLX-mediated RLX expression. NCI-N87 cells were treated with oAd/RLX at 0.2-5 MOI. The RLX gene expression level was determined by RT-PCR. (C) Masson's trichrome staining of PBS- or oAd/RLX- treated tumor tissue. The scale bar represents 1 mm. (D) Immunohistochemistry of collagen type I (brown) in NCI-N87 tumors treated with PBS or oAd/RLX. The scale bar represents 20 μm. (E) Quantification of collagen type I expression from the immunohistochemistry image. Data are presented as mean ± SD of five independent measurements; ***$P < 0.001$.

Jung B-K, *et al. J Immunother Cancer* 2020; 8:e000763. doi: 10.1136/jitc-2020-000763

Supplementary Figure 2

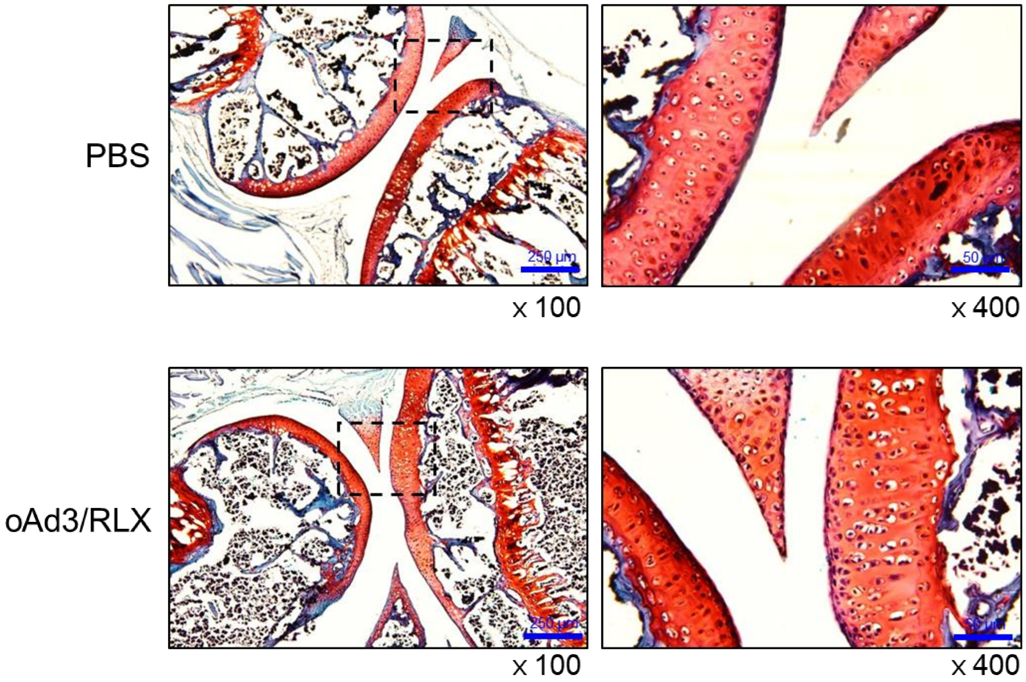

Fig. S2. Histological analysis of articular cartilage in the knee joint. Histological results of Safranin-O staining of articular cartilage in the knee joint of PBS- or oAd/RLX-treated mice. Original magnifications: ×100 or ×400. The scale bar represents 250 µm or 50 µm.

Supplementary Figure 3

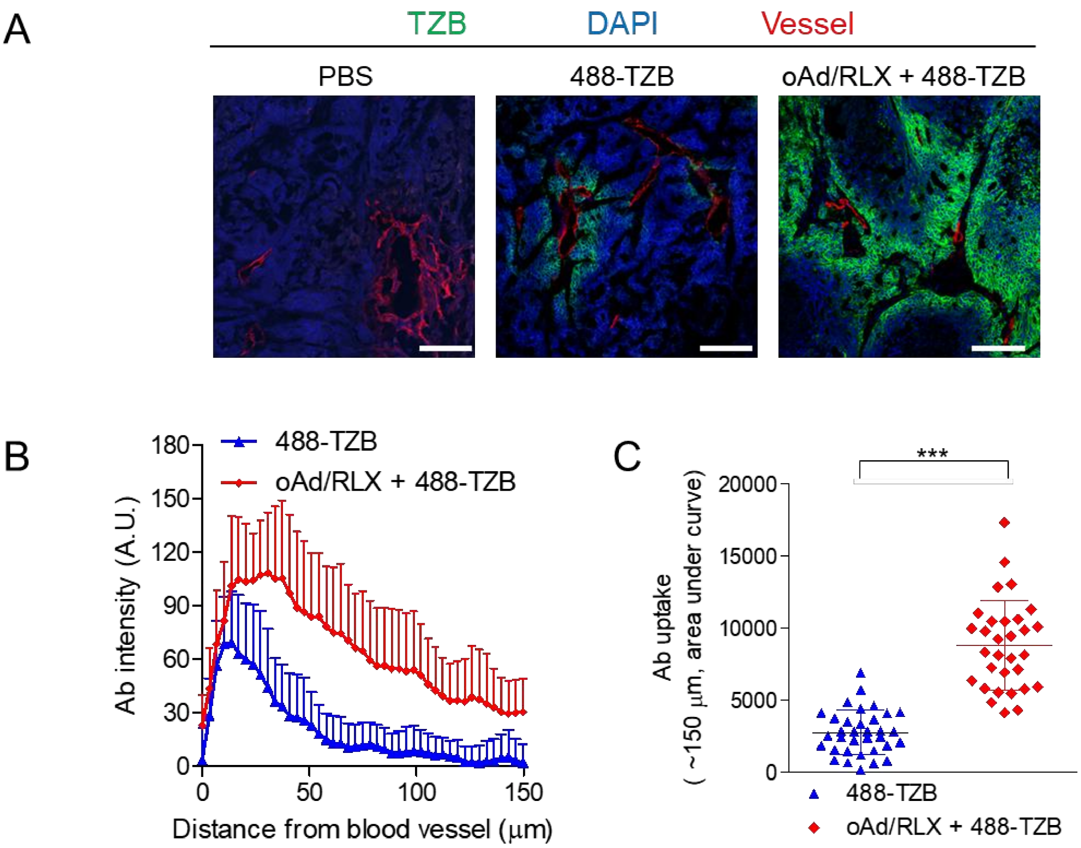

Fig. S3. Quantification of penetrated TZB from the blood vessel in tumor tissues. (A) Fluorescence images magnified from the white boxes labeled **β** in **Fig. 1A.** 488-TZB (green), rhodamine-lectin positive functional blood vessels (red), and DAPI-stained nuclei (blue) are shown for each group. Original magnification: ×100. The scale bar represents 100 μm. (B) Intensity profile of 488-TZB relative to the blood vessel (0-150 μm from blood vessel). (C) Uptake of 488-TZB quantified by area under curve analysis (0-150 μm from the blood vessel). Quantitative data are presented as mean optical density ± SD (n=10); ***P* < 0.01.

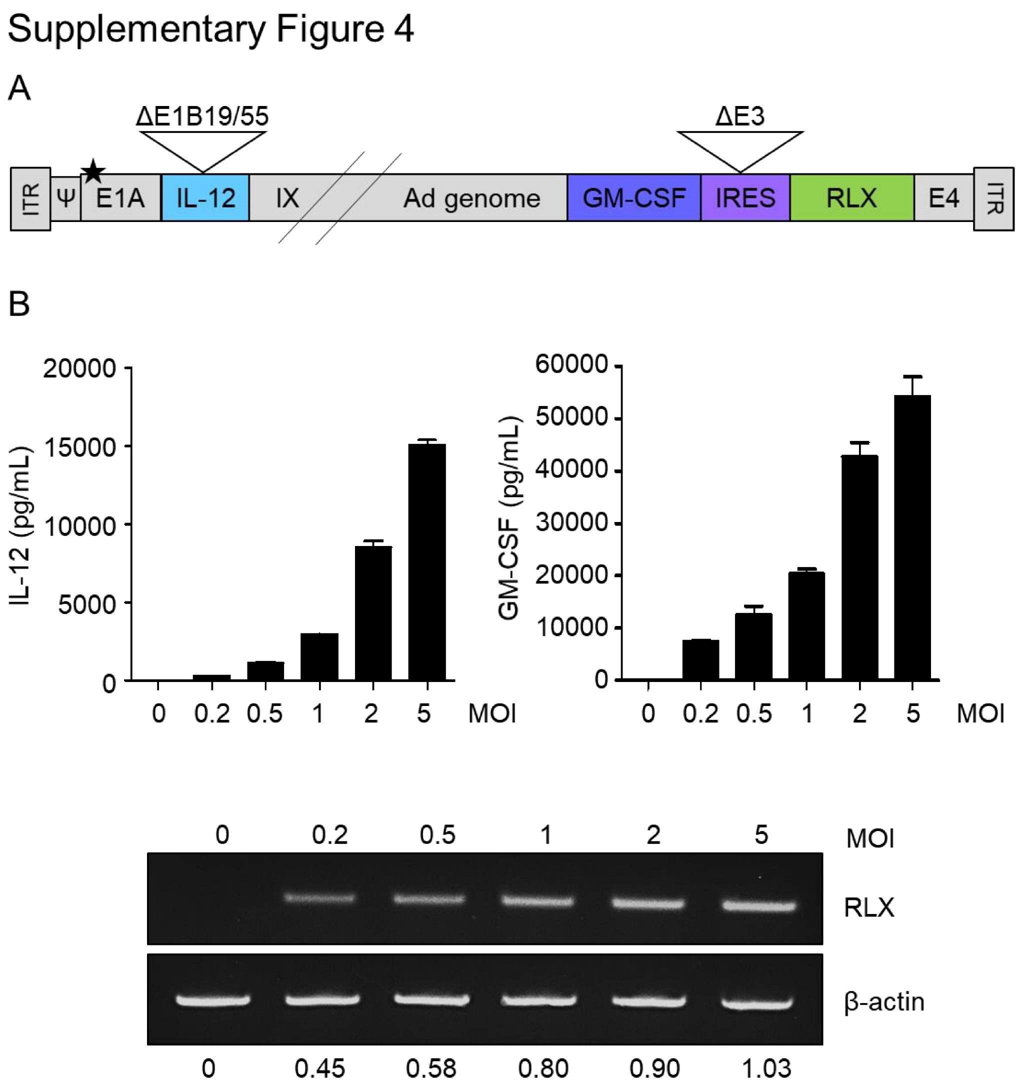

Fig. S4. Characterization of oAd/IL12/GM-RLX. (A) The oAd/IL12/GM-RLX construct. (B) Armed gene expression from oAd/IL12/GM-RLX. HaP-T1 cells were treated with oAd/IL12/GM-RLX at 0.2-5 MOI. Gene expression levels were determined by ELISA for IL-12 and GM-CSF or RT-PCR for RLX. Data are presented as mean ± SD (n=3).

Supplementary Figure 5

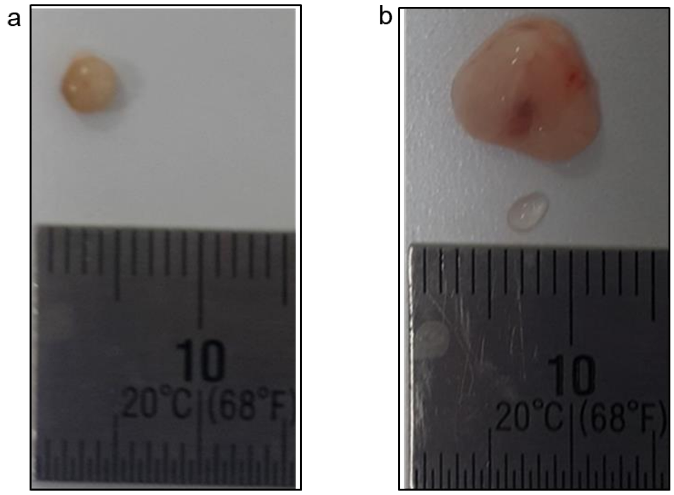

a : ^{64}Cu-αPD-1
b : oAd/IL12/GM-RLX + ^{64}Cu-αPD-1

Fig. S5. Representative photographs of DLNs. Representative DLNs from hamsters treated with (a) ^{64}Cu-αPD-1 or (b) oAd/IL12/GM-RLX plus ^{64}Cu-αPD-1. The DLN volume in the oAd/IL12/GM-RLX plus ^{64}Cu-αPD-1-treated group was increased by 48-fold compared with that in the ^{64}Cu-αPD-1-treated group

Jung B-K, *et al. J Immunother Cancer* 2020; 8:e000763. doi: 10.1136/jitc-2020-000763

Supplementary Figure 6

A

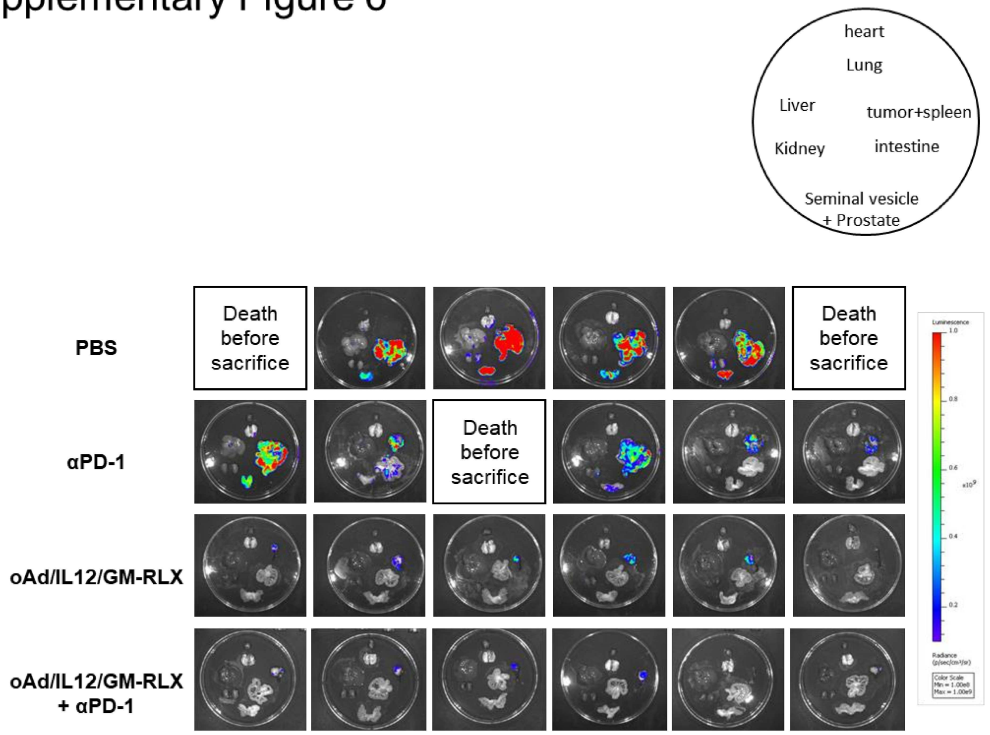

B

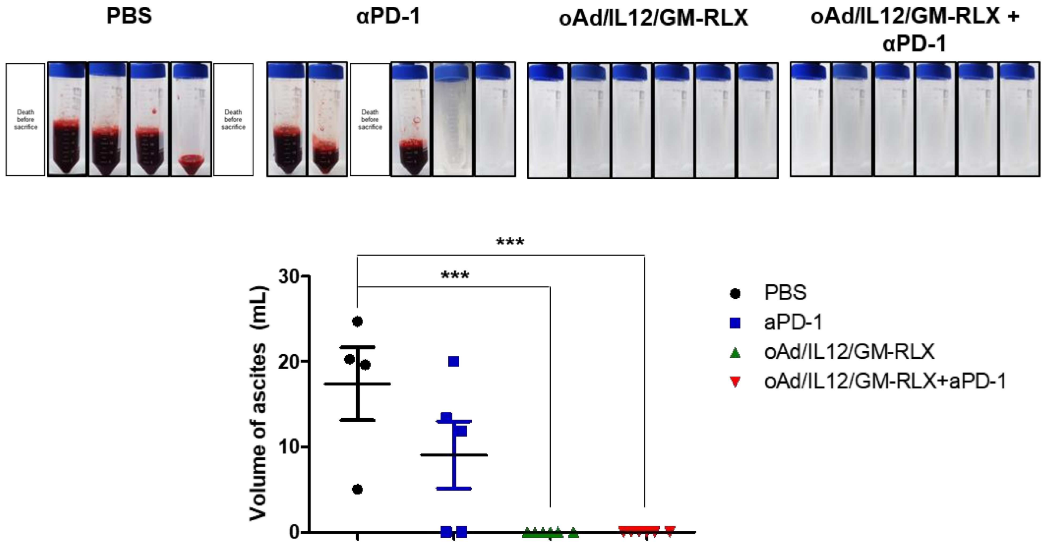

1

Fig. S6. Inhibition of metastasis by combination therapy oAd/IL12/GM-RLX with αPD-1 in hamster pancreatic orthotopic tumor model. The hamsters with orthotopically established HP-1 pancreatic tumors were injected oAd/IL12/GM-RLX and/or αPD-1 as described in **Fig. 4E**. (A) Individual Luciferase imaging in various organs (heart, lung, liver, kidney, spleen, intestine, seminal vesicle, and prostate) and tumor or (B) quantity of ascites of each treatment groups in luciferase-expressing HP-1 orthotopic pancreatic tumor model at 11 days post first treatment.

Supplementary Figure 7

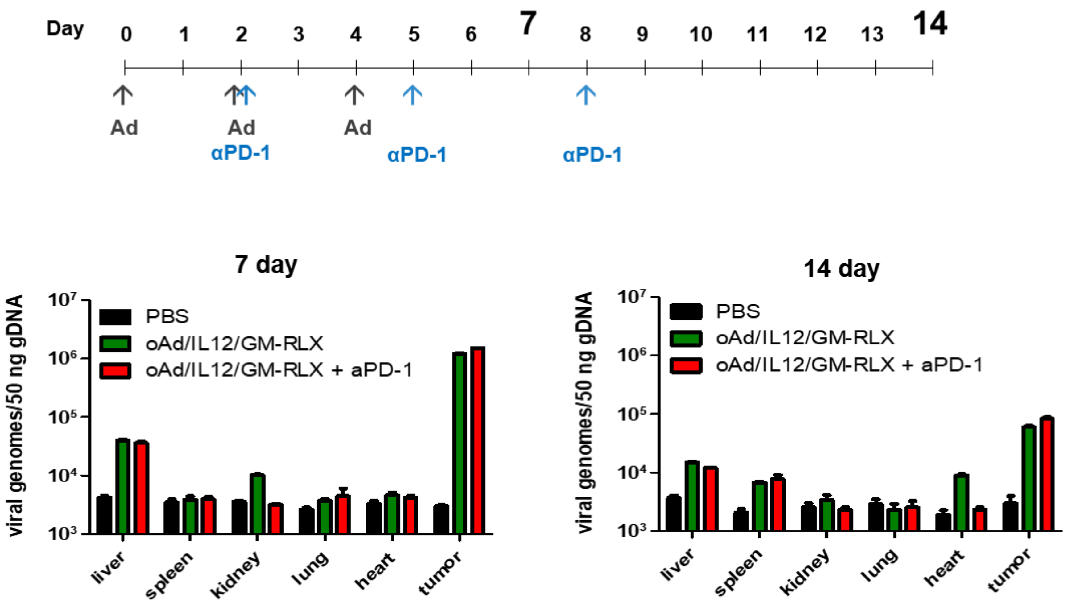

Fig. S7. Biodistribution study. HaP-T1 tumor-bearing hamsters were administered intratumorally with 7 × 10^7 VP of oAd/IL12/GM-RLX three times (day 0, 2, 4) and/or intraperitoneally with 10 mg/kg of αPD-1 three times (day 2, 5, 8), along with PBS as control. The liver, spleen, kidney, lung, heart, and tumor tissues were harvested at 7- or 14-day post initial virus treatment, and Q-PCR was performed to detect the viral genomes. Data are presented as mean ± SD (n=3).

Jung B-K, *et al. J Immunother Cancer* 2020; 8:e000763. doi: 10.1136/jitc-2020-000763

Supplementary Figure 8

	BUN (mg/dL)	Creatinine (mg/dL)	AST (U/L)	ALT (U/L)
PBS	20.3±0.6	≤ 0.20	53±2.0	52.7±0.6
αPD-1	20.7±0.6	≤ 0.20	59.3±1.2	80.3±1.2
oAd/IL12/GM-RLX	24.0±0.0	≤ 0.22	61.0±1.0	86.0±1.7
oAd/IL12/GM-RLX + αPD-1	22.7±0.6	≤ 0.20	49.0±1.0	52.0±1.0

Fig. S8. *In vivo* toxicity. HaP-T1 tumor-bearing hamsters were administered intratumorally with 7×10^7 VP of oAd/IL12/GM-RLX three times (day 0, 2, 4) and/or intraperitoneally 10 mg/kg of three times (day 2, 5, 8), along with PBS as control. The BUN, creatinine, AST, and ALT levels in serum were measured at 72 hr after last treatment. Data are presented as mean ± SD (n=3).

Supplementary Figure 9

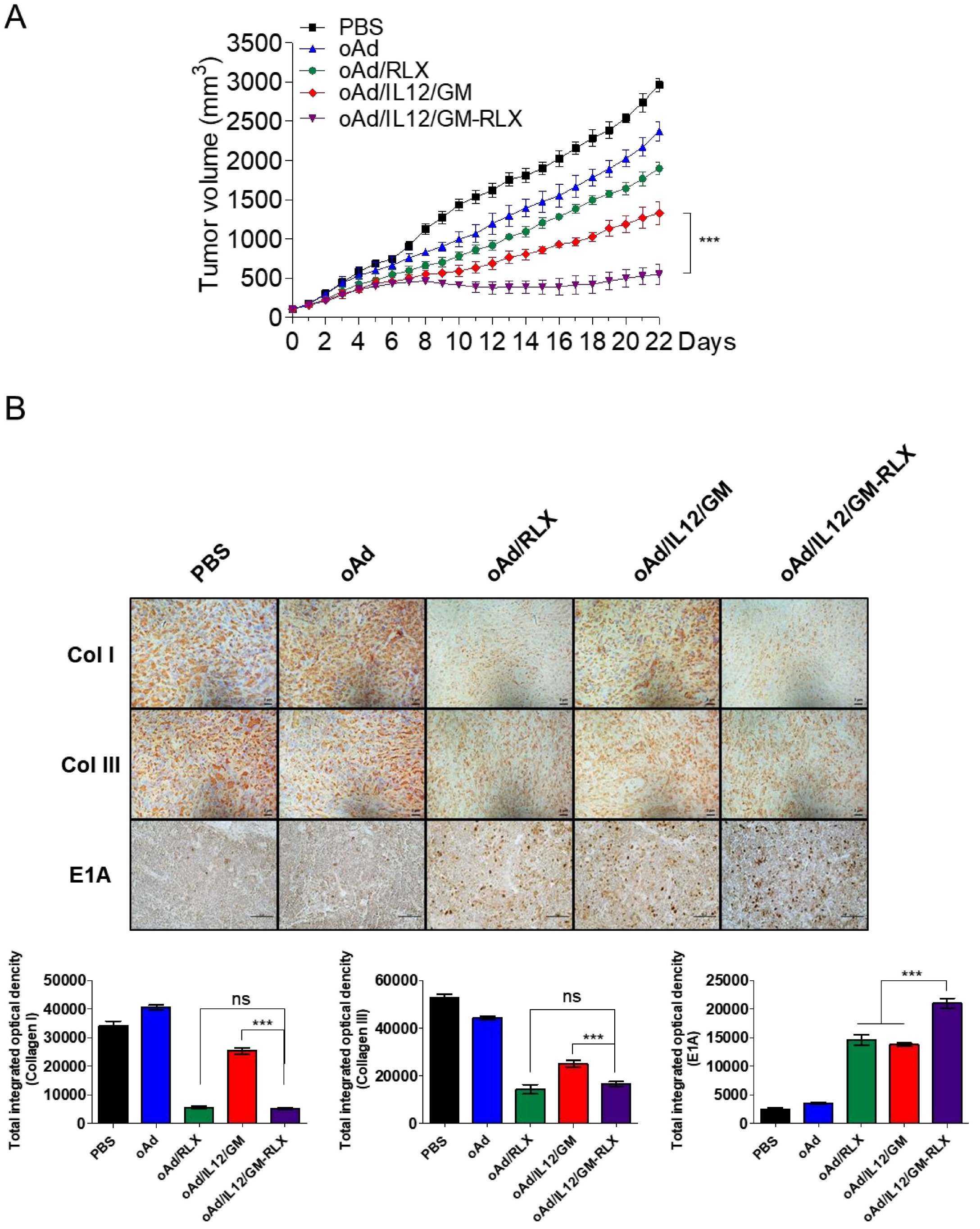

Supplementary Figure 9

c

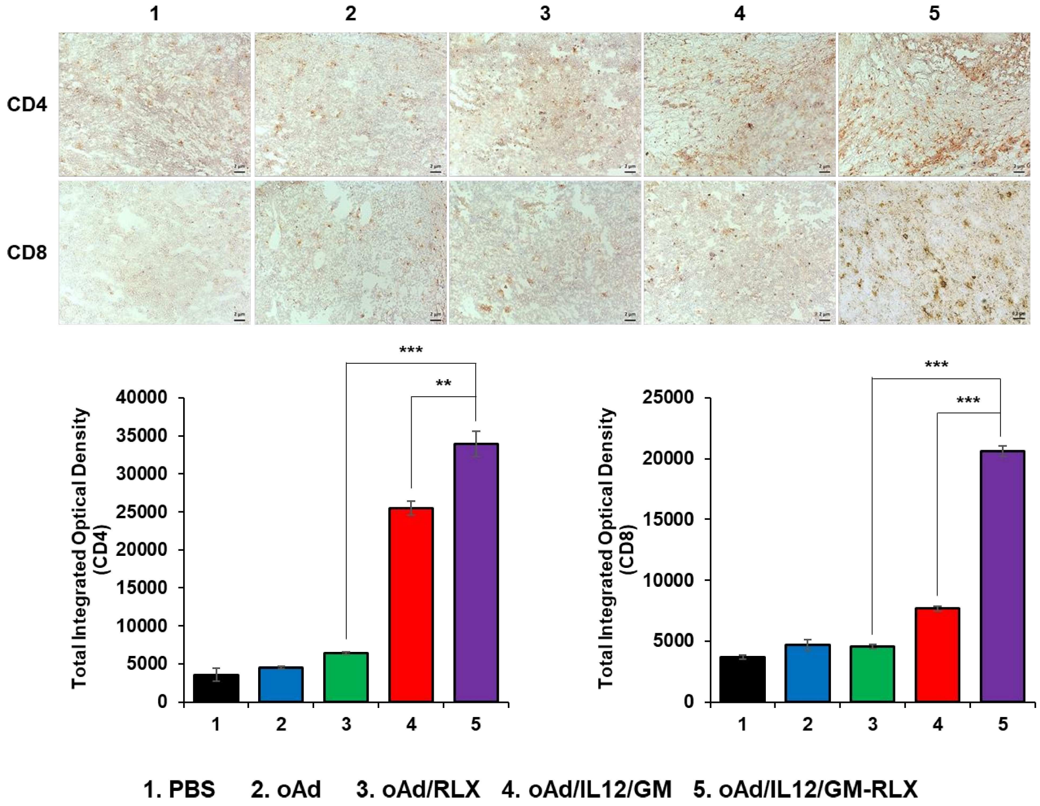

D

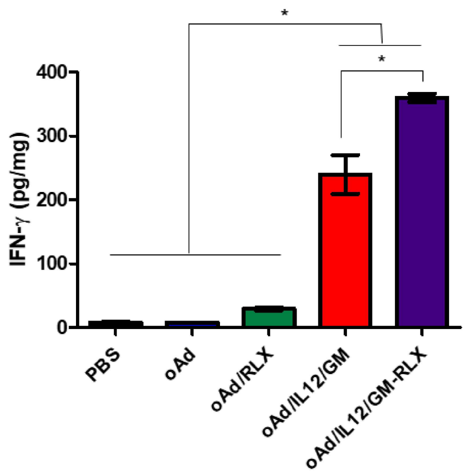

Fig. S9. Comparison of therapeutic efficacy of oAd/RLX, oAd/IL12/GM, and oAd/IL12/GM-RLX. Syrian hamsters were subcutaneously injected with HaP-T1 cells to establish pancreatic tumors. When the average tumor volume reached 90-100 mm^3, the tumors were injected with 1×10^8 VP of oAd, oAd/RLX, oAd/IL12/GM, and oAd/IL12/GM-RLX at day 0, 2, and 4. (A) The average tumor growth curves of HaP-T1 tumor bearing hamsters after treatment. The tumor volume was measured every day until the end of the study. Data are presented as mean ± SD (n=5); ***$P < 0.001$. (B, C) Immunohistochemical analysis. Tumor tissues were collected on day 7 after the first virus injection and stained with collagen I, collagen III, adenovirus E1A, CD4, and CD8 Abs. The representative images were displayed. The scale bar represents 2 μm (collagen I and collagen III, CD4 and CD8), or 50 μm (E1A). The positive signal was quantified with MetaMorph software (n=3); ***$P < 0.001$ or **$P < 0.01$ (D) IFN-γ expression in tumor tissues were analyzed by ELISA at 7 days post initial virus injection (n=3); *$P < 0.05$.

Table S1. Dosimetry of ^{64}Cu-TZB in tumors and organs

Organ or parameter	^{64}Cu-TZB (n=4)	oAd/RLX + ^{64}Cu-TZB (n=6)
Tumor	755.962 ± 126.974	1068.911 ± 200.635
Adrenals	0.013 ± 126.974	0.018 ± 0.0032
Brain	0.033 ± 0.010	0.036 ± 0.0115
Breasts	0.006 ± 0.001	0.009 ± 0.0018
Gallbladder wall	0.013 ± 0.002	0.016 ± 0.0032
Lower large intestine wall	0.005 ± 0.002	0.004 ± 0.0007
Small intestine	0.006 ± 0.002	0.006 ± 0.0014
Stomach wall	0.139 ± 0.038	0.164 ± 0.0463
Upper large intestine wall	0.006 ± 0.001	0.007 ± 0.0012
Heart wall	0.168 ± 0.024	0.236 ± 0.0689
Kidneys	0.010 ± 0.001	0.012 ± 0.0020
Liver	0.124 ± 0.002	0.147 ± 0.0341
Lungs	0.183 ± 0.011	0.258 ± 0.0398
Muscle	0.005 ± 0.001	0.006 ± 0.0010
Ovaries	0.007 ± 0.002	0.005 ± 0.0024
Pancreas	0.020 ± 0.002	0.026 ± 0.0048
Red Marrow	0.005 ± 0.002	0.006 ± 0.0011
Osteogenic	0.004 ± 0.042	0.005 ± 0.0009
Skin	0.002 ± 0.001	0.003 ± 0.0005
Spleen	0.502 ± 0.026	0.714 ± 0.0873
Thymus	0.011 ± 0.002	0.015 ± 0.0035
Thyroid	0.002 ± 0.019	0.003 ± 0.0005
Urinary Bladder	0.255 ± 0.034	0.191 ± 0.0891
Uterus	0.294 ± 0.001	0.169 ± 0.3622
Total body	0.014 ± 0.002	0.015 ± 0.005
ED Equivalent (mSv/MBq)	0.114 ± 0.032	0.131 ± 0.022
ED (mSv/MBq)	0.075 ± 0.011	0.090 ± 0.015

Table S1. Dosimetry of ^{64}Cu-Trastuzumab in tumors and organs. ^{64}Cu-TZB was intravenously injected with/without oAd/RLX into NCI-N87 tumor-bearing mice. The absorbed dose of ^{64}Cu-TZB to tumors and organs was measured at 40 hr post ^{64}Cu-TZB treatment.

Reference

1. Kim J, Kim PH, Nam HY, Lee JS, Yun CO, Kim SW: **Linearized oncolytic adenoviral plasmid DNA delivered by bioreducible polymers.** *J Control Release* 2012, **158:**451-460.
2. Choi KJ, Zhang SN, Choi IK, Kim JS, Yun CO: **Strengthening of antitumor immune memory and prevention of thymic atrophy mediated by adenovirus expressing IL-12 and GM-CSF.** *Gene Ther* 2012, **19:**711-723.
3. Jung KH, Choi IK, Lee HS, Yan HH, Son MK, Ahn HM, Hong J, Yun CO, Hong SS: **Oncolytic adenovirus expressing relaxin (YDC002) enhances therapeutic efficacy of gemcitabine against pancreatic cancer.** *Cancer Lett* 2017, **396:**155-166.
4. Kim JH, Lee YS, Kim H, Huang JH, Yoon AR, Yun CO: **Relaxin expression from tumor-targeting adenoviruses and its intratumoral spread, apoptosis induction, and efficacy.** *J Natl Cancer Inst* 2006, **98:**1482-1493.
5. Choi KJ, Kim JH, Lee YS, Kim J, Suh BS, Kim H, Cho S, Sohn JH, Kim GE, Yun CO: **Concurrent delivery of GM-CSF and B7-1 using an oncolytic adenovirus elicits potent antitumor effect.** *Gene Ther* 2006, **13:**1010-1020.
6. Lee YS, Kim JH, Choi KJ, Choi IK, Kim H, Cho S, Cho BC, Yun CO: **Enhanced antitumor effect of oncolytic adenovirus expressing interleukin-12 and B7-1 in an immunocompetent murine model.** *Clin Cancer Res* 2006, **12:**5859-5868.
